# Gene expression and functional annotation of human choroid plexus epithelium failure in Alzheimer’s disease

**DOI:** 10.1186/s12864-015-2159-z

**Published:** 2015-11-16

**Authors:** Arthur A. Bergen, Sovann Kaing, Jacoline B. ten Brink, The Netherlands Brain Bank, Theo G. Gorgels, Sarah F. Janssen

**Affiliations:** Department of Clinical Genetics, Academic Medical Centre, Amsterdam, AMC, Meibergdreef 9, 1105 AZ AMC Amsterdam, The Netherlands; The Netherlands Institute for Neurosciences (NIN-KNAW), Amsterdam, The Netherlands; University Eye Clinic Maastricht, MUMC, Maastricht, The Netherlands; Department of Ophthalmology, VUMC, Amsterdam, The Netherlands

## Abstract

**Background:**

Alzheimer’s disease (AD) is the most common form of dementia. AD has a multifactorial disease etiology and is currently untreatable. Multiple genes and molecular mechanisms have been implicated in AD, including ß-amyloid deposition in the brain, neurofibrillary tangle accumulation of hyper-phosphorylated Tau, synaptic failure, oxidative stress and inflammation. Relatively little is known about the role of the blood-brain barriers, especially the blood-cerebrospinal fluid barrier (BCSFB), in AD. The BCSFB is involved in cerebrospinal fluid (CSF) production, maintenance of brain homeostasis and neurodegenerative disorders.

**Results:**

Using an Agilent platform with common reference design, we performed a large scale gene expression analysis and functional annotation of the Choroid Plexus Epithelium (CPE), which forms the BCSFB. We obtained 2 groups of freshly frozen Choroid Plexus (CP) of 7 human donor brains each, with and without AD: Braak stages (0–1) and (5–6). We cut CP cryo-sections and isolated RNA from cresyl-violet stained, laser dissected CPE cells. Gene expression results were analysed with T-tests (R) and the knowledge-database Ingenuity.

We found statistically significantly altered gene expression data sets, biological functions, canonical pathways, molecular networks and functionalities in AD-affected CPE. We observed specific cellular changes due to increased oxidative stress, such as the unfolded protein response, E1F2 and NRF2 signalling and the protein ubiquitin pathway. Most likely, the AD-affected BCSFB barrier becomes more permeable due to downregulation of CLDN5. Finally, our data also predicted down regulation of the glutathione mediated detoxification pathway and the urea cycle in the AD CPE, which suggest that the CPE sink action may be impaired. Remarkably, the expression of a number of genes known to be involved in AD, such as APP, PSEN1, PSEN2, TTR and CLU is moderate to high and remains stable in both healthy and affected CPE. Literature labelling of our new functional molecular networks confirmed multiple previous (molecular) observations in the AD literature and revealed many new ones.

**Conclusions:**

We conclude that CPE failure in AD exists. Combining our data with those of the literature, we propose the following chronological and overlapping chain of events: increased Aß burden on CPE; increased oxidative stress in CPE; despite continuous high expression of TTR: decreased capability of CPE to process amyloid; (pro-) inflammatory and growth factor signalling by CPE; intracellular ubiquitin involvement, remodelling of CPE tight junctions and, finally, cellular atrophy. Our data corroborates the hypothesis that increased BCSFB permeability, especially loss of selective CLDN5-mediated paracellular transport, altered CSF production and CPE sink action, as well as loss of CPE mediated macrophage recruitment contribute to the pathogenesis of AD.

**Electronic supplementary material:**

The online version of this article (doi:10.1186/s12864-015-2159-z) contains supplementary material, which is available to authorized users.

## Background

Alzheimer’s disease (AD) is the most common form of dementia affecting 35 million people world-wide. It is a complex disease caused by environmental and genetic risk factors. Treatment is currently not available [1]. Genetic analysis of families with rare, early onset AD implicated a few disease genes, such as APP, PSEN1 and PSEN2 [2]. Recently, additional rare variants in APP, TREM2 modulating the sporadic, late onset AD disease phenotype were discovered [3, 4]. The most recent large scale DNA analyses implicated, apart from the APOE locus, 19 loci containing common, small-effect, susceptibility variants in over 100 AD candidate disease genes, such as CR1, CLU, PICALM, BIN1, EPHA1, MS4A, CD33, CD2AP and ABCA7 [5–8]. Pathobiological pathways such as insulin signalling, synaptic or mitochondrial failure, oxidative stress, inflammation, vascular effects and cholesterol metabolism have been implicated in AD [1]. However, the most important pathobiological hallmarks of AD are the progressive extracellular accumulation of ß amyloid peptide (Aß) in the brain and the intra-neuronal accumulation of Aß and tangles composed of hyperphosphorylated Tau protein. Extracellular overproduction of Aß, insufficient Aß-processing or too little Aß clearance from the brain leads (initially) to higher levels these peptides in the CSF, extracellular plaques and neuronal dysfunction or death [1].

Studies on the involvement of the blood brain-barriers in AD disease were primarily focused on the (increased) permeability of the endothelial BBB barrier and the potential deficient clearance of (excess) Aß peptides across it [9]. The endothelial (inner) BBB is sealed by tight junction complexes made up by claudines, zonula occludens and junctional adhesion proteins, which do not exist in the “leaky” peripheral circulation. The endothelial cells of the BBB are surrounded by a basement membrane, in which pericytes reside. Within the brain, the BBB capillaries are surrounded by the glia limitans, which consist of linked astrocyte end feet. All these cell types contain a variety of peptidases and cholinesterases. Consequently, any (bio-) molecule, including actively transported Aß, has to overcome several physical and enzymatic hurdles in order to cross the BBB from the blood to the brain parenchyma, or vice versa [10–12].

In contrast with the endothelial BBB barrier, the potential role of the Blood-CSF (BCSFB) barrier in AD has only recently drawn considerable attention. The epithelial (outer) BCSFB barrier is located at the choroid plexus (CP). The CPs protrude as plated or lobular structures into all brain ventricles. They consist of folded monolayers of polarized neuro-epithelial cells (CPE) as an extension of the ependymal cell layer, and envelop a vascularized stroma. The cuboidal layer of CPE cells, linked together by tight junctions, forms the BCSFB barrier [13]. Unlike the micro-vessels at the BBB barrier, capillaries in the CP stroma are fenestrated. Thus, neither endothelial cells nor a local glia limitans structure form an obstacle for biomolecule transport over the BCSFB barrier. The main functions of the CPE are the production of CSF, transport or production of a number of (bio) molecules, and to act as a sink for a number of CSF waste products [13].

Chalbot et al. [14] pointed out that CPE damage may be among the first signs of AD, at least in a subset of patients. Indeed, anatomical, physiological, biochemical and immunological evidence exists that the CP is involved in onset or progression of AD. The morphological and physiological changes of the CP in AD resemble those of strongly accelerated aging: AD-affected CPE cells show progressive atrophy and accumulate numerous lipofuscin vacuoles. Compared to age-matched controls, the AD affected CP contains increased amounts of psammoma bodies [15, 16].

These structural changes are accompanied by mitochondrial dysfunction, oxidative stress and increased cell death, which strongly affect the secretory function of the CP. Silverberg (2003) suggested that the CSF pressure, primarily maintained by the CP, is first normal, than slowly rises in early stages of AD, and subsequently drops again in late stages of the disease [17]. In AD, not only water production into the brain, but also CPE production or transport of a range of (bio-) molecules may be compromised. These molecules include ions, vitamins, growth factors and hormones [13]. Elegant studies on the CP in AD in *in vitro* and in *in vivo* models suggested that the CP takes up (excess) Aß40 and Aß42 peptides from the CSF and may clear them from the CNS via LRP1 and other transporters [18, 19]. Interestingly, the CP uniquely produces and secretes transthyretin (TTR) [20], a tetrameric protein that binds and stabilizes soluble Aß peptides in CPE cells and the CSF. Indeed, decreased TTR levels in the CSF were previously correlated with dementia and AD [21], although these findings are not unchallenged [22]: A recent literature review did not support CP failure in human subjects with AD or elderly with regards to production, transport or secretion of TTR, vitamin C, folate, and ions [22].

The CP may also be involved in neuro-inflammation: Initially, degeneration of neurons in the AD affected brain lead to a local pro-inflammatory immune cell response of activated resident glia cells. Signalling by these locally activated glia cells, via the CSF, may activate the CPE at the CSFB barrier to recruit CPE resident or peripheral immune cells. Peripheral inflammation-resolving macrophages cross, in part, the CP. Interestingly, the fate of the local inflamed tissue (death or restoration) in the brain may thus depend on (1) the ability of the CP to transform parenchyma derived signals into other peripheral macrophages recruitment signals, or (2) the barrier properties of the CP with respect to inflammation-resolving macrophages migrating through the barrier [23].

Most interestingly, recent studies suggests that the CP also participates in the neural stem cell proliferation and differentiation, most likely through secretion of growth factors and signaling molecules which can reach, via the CSF, the sub-ventricular zone [24]. Finally, Bolos and co-workers [25] very recently showed that healthy choroid plexus implants rescued AD linked pathologies in animal models. These findings highlight the importance of (a healthy) choroid plexus in AD. In the current study, we investigate the human transcriptome differences between the CPE of healthy and AD-affected individuals. Our data suggest that CPE damage strongly contributes to AD pathology.

## Methods

### Ethics statement

This study was performed after institutional approval of the Netherlands Institute for Neuroscience (NIN-KNAW), Amsterdam, the Netherlands. The human CP material was obtained from the Netherlands Brain Bank (NBB; Amsterdam, The Netherlands). In accordance with the international declaration of Helsinki, the NBB obtained written permission from the donors for brain autopsy and the use of brain material and clinical information for research purposes. All human data were analysed anonymously.

### Gene expression of healthy and affected human CPE

Human CPE samples were obtained from donor brains by a team of expert neuro-pathologists of the NBB. We used freshly frozen human CPE samples from lateral ventricles of 7 healthy (Braak 0–1; abbreviated as (Br0–1)), and 7 AD-affected (Braak 5–6; (Br5–6)) [26] The donor age’s varied between 51 and 73 years and the post-mortem times between 3 and 10 h. ApoE genotypes are available. All CPE donors were healthy (controls) or had no history of other brain diseases than AD (Additional file 1: Table S1).

For a detailed description of our microarray studies, including (limitations and strengths of) study design, sampling, staining, laser dissection microscopy, RNA isolation and stringent quality control, single amplification, labelling and hybridization procedures, see our previous publications of Booij et al. (2009) [27] and Janssen et al. (2012) [28]. We followed MIAMI guidelines and submitted all our gene-expression data to the GEO database Submission no. GSE61196 [NCBI tracking system #17129928].

In short, we used a study design with RNA from human RPE/choroid as common reference, as described [28]. We selectively laser captured CPE cells out of 10 μm cryosections. CPE cells could be easily visualized using immunosytaining with anti-TTR and ZO-1 antibodies (Additional file 2: Figure S1), and histological stainings such as with Cresyl-violet. After isolation and quality controls, RNA was amplified once and then labeled and hybridized against human 4x44K catalogue microarrays (Agilent Technologies, Amstelveen, The Netherlands).

The microarray image files were subsequently analysed and processed by Agilent Feature Extraction Software (Agilent Technologies) and log2 mean intensities were assigned to the spots. Next, we normalized the mean expression/intensity data of each individual sample against the common reference sample in the computer program R (method ‘aquantile’; version 2.14.0 for Windows, R Development Core Team, 2009).

### Statistical analysis and functional annotation

Our microarray analysis covered seven samples of healthy human CPE (Br0–1) and seven samples of AD-affected CPE (Br5–6). We used the normalized expression data of each individual sample for a statistical T-test in R (package LIMMA; including BH correction for multiple testing) to investigate statistically significant gene expression differences between the healthy and AD-affected CPE disease state (described in Results section).

We selected and analysed two data-sets: Genes whose expression was upregulated in AD CPE and genes downregulated in AD CPE. After the T-test, the resulting data-sets were processed, *before functional annotation*, as follows: Final selections were made of genes (expression changes) with p < 0.05, fold change (FC) > 2.0. Potential technical duplicates present on the array, which might otherwise skew functional interpretation, were removed. Functional annotation was carried out with the knowledge database Ingenuity (www.ingenuity.com). The core analysis of Ingenuity delivers significantly enriched themes for the input dataset; in terms of biological functions, canonical pathways and molecular networks. The molecular networks are either (combinations of) functional molecular networks (in which function between entries is the main “glue” between molecules) or structural molecular networks (those networks which cluster around certain entries, such as APP or UBC, primarily through experimentally proven physical interactions between the (predicted) proteins. For the core analysis Ingenuity standard settings were used. All non-modified Figures and Tables derived from Ingenuity are subject to copyright owned by Qiagen Inc.

### Immuno-histochemistry

Immuno-histochemistry was done on 6 μm thick cryo-sections of CP from healthy donors and from donors diagnosed with AD (Braak stage 6). Cryostate sections were fixed for 10 min in 4 % paraformaldehyde in 0.1 M phosphate buffer. For staining we employed standard procedures. CLDN5 was detected with Cy3 coupled secondary antibodies after labeling with anti-Claudin 5 antibody ab53765 (Abcam). Double staining for Transthyretin (TTR) and the tight junction protein 1 (ZO-1) was done using the Anti-TTR/Transthyretin Antibody (clone 10E1) IHC-plus™ LS-B2864, from LSBio, visualized with a Cy3 coupled secondary antibody (goat anti mouse, Jackson ImmunoResearch), and the ZO-1 antibody (Zymed 61-7300), visualized with Alexa 647 coupled secondary antibody (donkey anti rabbit, Jackson ImmunoResearch). Slides were photographed on a Leica SP8 confocal microscope.

### CPE molecular functional networks; AD disease labels from the literature

Finally, we also compared our molecular functional networks of the CPE with (lists of) AD genes/proteins derived from the scientific literature. We compiled these AD literature lists (Additional file 3: Table S2) by hand, via PubMed, or we used the “functions and disease” entry of Ingenuity. We labelled our data driven CPE molecular networks (Additional file 4: Figure S2 and Additional file 5: Figure S3) with entries from these lists. The most obvious CP disease label in this context is, of course, AD, but we independently compiled also gene lists for Multiple Sclerosis (MS) and Parkinson disease (Additional file 3: Table S2).

Previous literature studies also suggested that *CPE function* can be influenced or measured by Aß [29], Aß removal (via Transthyretin (TTR); APO-J, gelsolin, cystatin C, PTGDS, IGF1-2, AAT, megalin) [30], oxidative phosphorylation [31], mitochondrial respiratory chain defects (cytochrome C oxidase) [20, 32], Aß transport (Pgp, LRP1, LRP2), ascorbic acid (AA) and folate transport from the blood (FRα, SLC46A1 and SLC19A1) [22], CSF ion (iron, copper, zinc) [30] and pH homeostasis (Na-K-Cl cotransporter), secretion of growth factors, CSF production, as well as the immune function of the CPE during degenerative disease (TLRs, cytokine receptors, TNFαR, ICAM1, VCAM1, CXCL10, CD73 [23]. All these entries were also included in Additional file 3: Table S2 and used, double blind, to bio-informatically label relevant parts of our data-driven CPE molecular networks (Additional file 4: Figure S2 and Additional file 5: Figure S3).

## Results

### Gene expression upregulated in AD-affected CPE

**Fig. 1:**
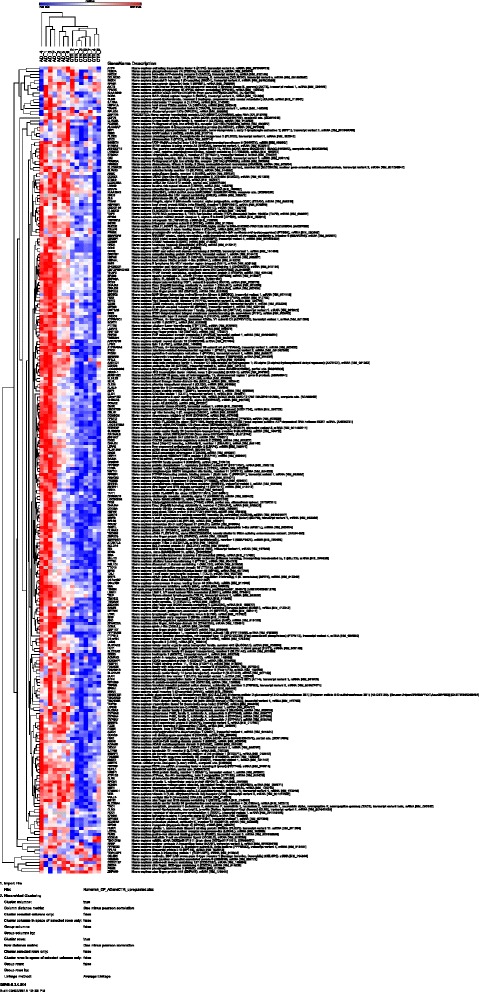
Cluster analysis of mean expression values for the up-regulated genes in AD (Br5–6) choroid plexus compared to healthy control choroid plexus (Br0–1). Cluster analysis was performed using GENE-E (version 3.0.204; analysis performed on 10/10/2015) with the “one minus Pearson correlation” and the “Average linkage method”. See also http://www.broadinstitute.org/cancer/software/GENE-E/. On the Y-axis gene names are shown, on the X-axis the individual samples. Blue and red colors indicate, respectively, relatively low and high numerical expression values. The dendograms at the top and the left side denote the (close) relationships between, respectively, the samples and the genes. Analysis parameter values are given below the figure. Both the AD and control samples cluster nicely together. Please use your PDF zoom function to evaluate details.

In total, we found 343 upregulated genes in (Br5–6) AD-affected CPE ((BH P > 0.05; FC > 2.0; Additional file 6: Table S3). The most highly upregulated individual genes were: LSMEM1, ETNPPL, HSAPA1A/B and 6, CELSR1, RCC1, DNAJB1, SERPINC1, ZBTB1 and ZFAND2A (Table 1). We constructed a data driven heatmap of the 343 upregulated genes, which shows the correlation of gene expression among the samples used. Indeed, the heatmap reveals two clusters of CPE control and AD-affected CPE samples (Fig. 1). Ingenuity core analysis of the 343 upregulated genes assigned overrepresented biological pathways, canonical pathways, and functional molecular networks to our dataset.

**Table 1:** The top 10 most down- , and the top 10 most up- regulated (expression of) genes in AD affected CPE versus healthy CPE

Fold change	Symbol	Entrez gene name	Location
−18.03	C6orf58	Chromosome 6 open reading frame 58	Cytoplasm
−8.09	BRCA2	Breast cancer 2, early onset	Nucleus
−7.71	FABP4	Fatty acid binding protein 4, adipocyte	Cytoplasm
−7.18	RPS4Y1	Ribosomal protein S4, Y-linked 1	Cytoplasm
−6.57	PRND	Prion protein 2 (dublet)	Pl. Membr.
−6.27	LRRC74B	Leucine rich repeat containing 74B	Other
−6.26	C10orf107	Chromosome 10 open reading frame 107	Other
−5.76	NEBL-AS1	NEBL antisense RNA 1	Other
−5.66	RBM3	RNA binding motif (RNP1, RRM) protein 3	Cytoplasm
−5.39	TXLNGY	Taxilin gamma pseudogene, Y-linked	Other
11.37	LSMEM1	Leucine-rich single-pass membrane protein 1	Cytoplasm
9.45	ETNPPL	Ethanolamine-phosphate phospho-lyase	Other
9.15	HSPA1A/HSPA1B	Heat shock 70kDa protein 1A	Cytoplasm
7.04	HSPA6	Heat shock 70kDa protein 6 (HSP70B’)	Nucleus
6.56	LHFPL3-AS2	LHFPL3 antisense RNA 2	Other
6.24	CELSR1	Cadherin, EGF LAG seven-pass G-type receptor 1	Pl. Membr.
5.72	RCC1	Regulator of chromosome condensation 1	Cytoplasm
5.55	DNAJB1	DnaJ (Hsp40) homolog, subfamily B, member 1	Nucleus
5.42	SERPINC1	Serpin peptidase inhibitor, clade C (antithrombin), member 1	Extracell. Sp.
5.1	ZBTB1	Zinc finger and BTB domain containing 1	Nucleus

Names of the genes are abbreviated according to Genbank. For potential relationship with AD according to the scientific literature, see description in the main text

### Biological pathways

**Fig. 2:**
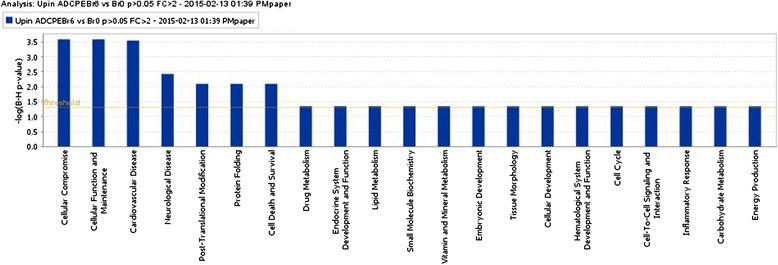
Graph of the most significant over-represented and upregulated biological pathways in (Br5-6) AD CPE. The height of the bars indicates the level of statistical significance. The straight horizontal yellow line represents the cut off value of statistical significance. The yellow line jumping up and down indicates the ratio of the number of genes present in the experimental dataset, divided by the theoretical number of genes that together would make up the functionality

Several biological pathways were identified in AD (Br5–6) CPE, but not in healthy CPE. Some of these pathways also occur in cardiovascular, neurological, inflammatory, hematological and connective tissue disorders. Functions assigned on a molecular and cellular level “more prominently present in (Br5–6) CPE than in (Br0–1) CPE” included: cellular compromise, cellular function and maintenance, post-translational modifications, protein folding as well as cell death and survival. More prominent physiological functions assigned to our upregulated gene expression dataset were: endocrine system development, embryological development, tissue morphology, hematological system development and function as well as increased immune cell trafficking (Fig. 2).

### Canonical pathways

**Fig. 3:**
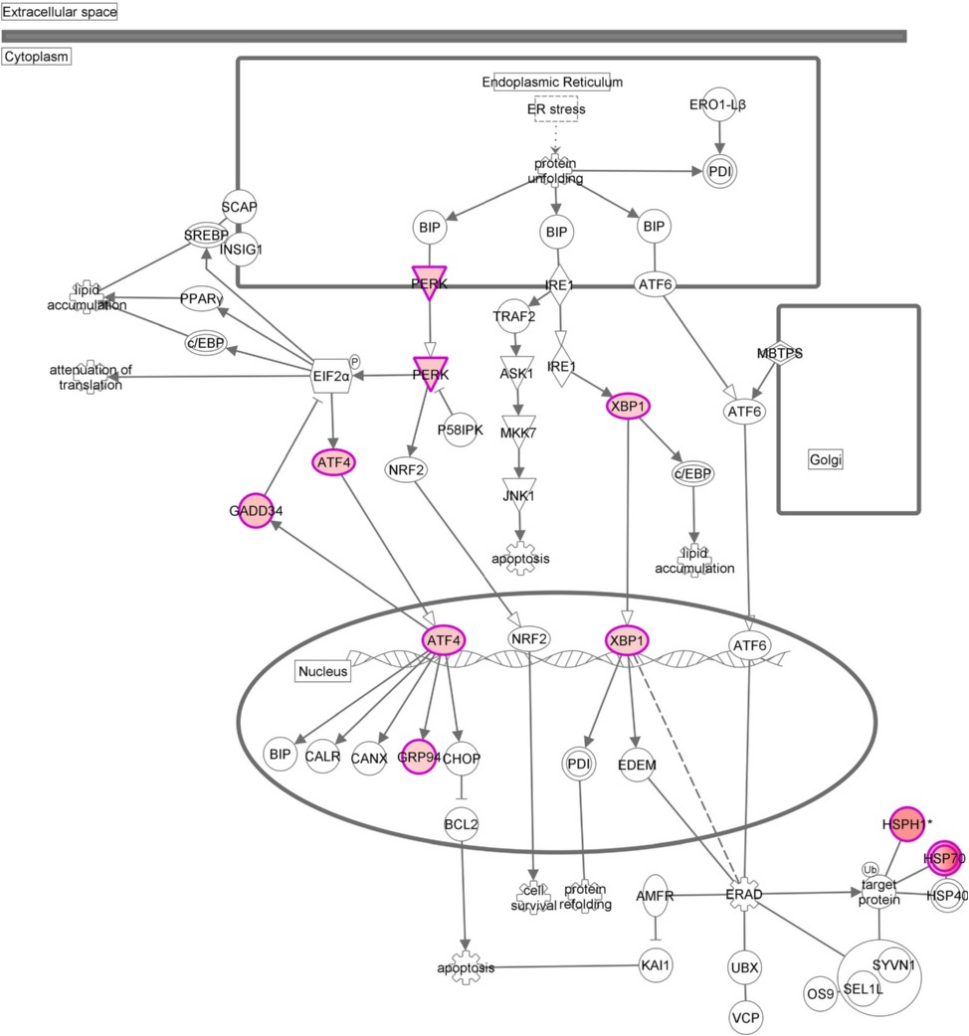
Upregulation of the over-represented unfolded protein response in AD affected CPE. In the schematic drawing all regulated potential molecular players of the unfolded protein response are presented. The filled (red) symbols represent the entries which are actually statistically significantly upregulated in AD affected CPE (Br5–6) in our data set(s). Other entries may also be expressed by the CPE, but are not regulated between healthy and AD-affected CPE

The most important canonical pathways suggested that cellular stress in AD (Br5–6) CPE is increased. The most important functional annotations assigned to the upregulated pathways in our dataset were: The unfolded protein response (Fig. 3), the endoplasmatic reticulum stress pathway, aldosterone signalling in epithelial cells, eNOS signalling and RAN signalling (summarized in Additional file 7: Figure S4).

### Functional molecular networks

Ingenuity analysis yielded 25 functional molecular networks. Only the most relevant networks are presented (Additional file 4: Figure S2), which also yielded literature labels related to AD. Functional annotation of the networks largely overlaps with those of the canonical pathways.

### Gene expression downregulated in AD-affected CPE

**Fig. 4:**
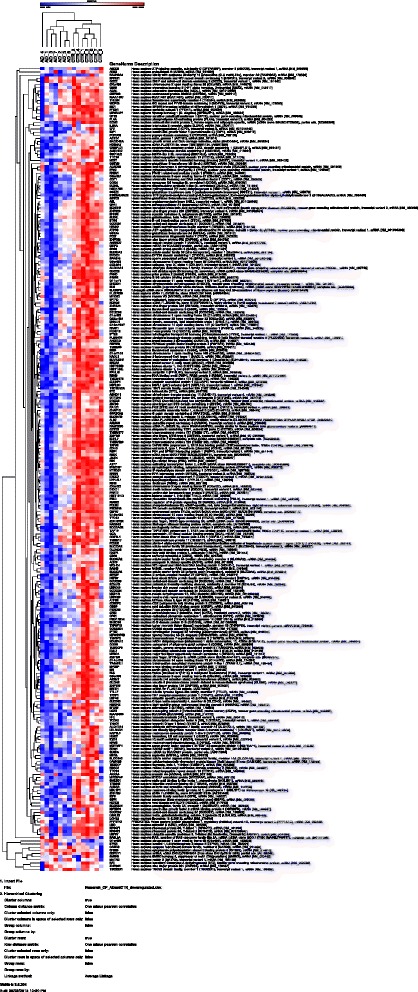
Cluster analysis of mean expression values for the down-regulated genes in AD (Br5–6) choroid plexus compared to healthy control choroid plexus (Br0–1). Cluster analysis was performed using GENE-E (version 3.0.204; analysis performed on 10/10/2015) with “one minus Pearson correlation” and “Average linkage method” http://www.broadinstitute.org/cancer/software/GENE-E/. On the Y-axis the gene symbols are shown, on the X-axis the individual samples. Blue and red colors indicate, respectively relatively low and high numerical expression values. The dendograms at the top and the left side denote the (close) relationships between, respectively, the samples and the genes. Analysis parameter values are given below the figure. Both the AD and control samples cluster nicely together. To view details, please use your PDF zoom function.

We performed a t-Test between our healthy human CPE (Br0–1) and AD (Br5–6) CPE gene expression data-sets (p < 0.05; BH corrected; FC > 2.0). We found 387 statistically significant entries downregulated in AD affected CPE (Additional file 8: Table S4). We constructed a data driven heatmap of these 387 downregulated genes, which shows the correlation of gene expression between the samples used. Indeed, the heatmap revealed two clusters of CP control and AD affected samples (Fig. 4). The top ten most downregulated genes in (Br5–6) CPE vs (Br0–1) CPE were: BRCA2, FABp4, RPS4Y1, PRND, C10orf107, RBM3, SHISA3, SLC34A2, RGS22, PRDM16 (Table 1). Functional annotation of the 387 genes revealed statistically significantly overrepresented biological functions, canonical pathways and functional molecular networks.

### Biological pathways: down in AD (Br5–6) affected CPE

The most prominent overrepresented gene expression features in our dataset pointed toward (changes in) basic cellular functions and disease. In terms of disease, Ingenuity recognized similar patterns/molecular mechanisms underlying both hereditary and developmental disorders as well as underlying ophthalmic and neurological disorders. The most important downregulated molecular and cellular functions were: cellular development, growth and proliferation, carbohydrate metabolism, dug metabolism and molecular transport. Most significantly, major changes in (Br5–6) CPE, compared to (Br0–1) CPE, were found in terms of embryonic/neural development and function as well as tissue development and morphology.

### Canonical pathways: down in (Br5–6) CPE

Functional annotation of our dataset implicated down regulation of the following canonical pathways in (Br5–6) CPE compared to (Br0–1) CPE: Glutathione mediated detoxification, LPS/IL1 mediated inhibition of RXR function, the urea cycle, dopamine and histamine degradation (Additional file 9: Figure S5).

### Molecular functional networks of down-regulated genes in (Br5–6) CPE

We present the most relevant selection of functional molecular networks of genes down-regulated in (Br5–6) AD CPE. We show only those networks which also received AD- literature labels. Obviously, the annotation of the canonical pathways and the molecular networks overlap (Additional file 5: Figure S3).

### CPE function or pathology, and AD literature labels

We labelled our data-driven functional molecular networks with relevant entries known from the literature (Additional file 3: Table S2, Additional file 4: Figure S2 and Additional file 5: Figure S3). We only present those networks which received also AD literature labels. Interestingly, multiple networks were labelled with multiple “AD” literature entries. This did not only, posteriorly, validate our dataset further, but also showed that defined molecular networks previously implicated in AD are (also) activated in CPE cells. Oxidative stress labels, CSF labels and iron, copper literature labels were assigned several times in the networks. In (Br5–6) CPE stages, a number of networks center on Ubiquitin C, indicating an important role for the ubiquitin system in the pathogenesis of AD affected CPE (See Additional file 4: Figure S2 and Additional file 5: Figure S3).

### Individual gene expression changes and quality control microarray data

To validate the (technical) outcome of our current gene expression studies, we performed several confirmatory RT-PCRs in triplicate as a quality control. Overall, and similar to the results in our previous neuro-epithelial microarray studies [27, 28, 33–35] we found a very good match between the microarray and the qPCR results. The results are summarized in Table 2.

**Table 2:** Validation of microarray analysis by RT-PCR (three independent repeats) of 17 selected genes

	RT-PCR#1		RT-PCR#2		RT-PCR#3		RT-PCR	Microarray	
Gene	F.C.	P value	F.C.	P value	F.C.	P value	Mean F.C.	Mean F.C.^a^	P value*
*AGXT2L1*	21.19	0.003	17.36	0.010	15.57	0.005	18.04	9.45	0.001
*SPP1*	5.92	0.004	6.59	0.002	8.09	0.001	6.87	4.16	0.040
*ITGAV*	3.21	0.004	3.20	0.026	3.33	0.011	3.25	3.43	0.002
*ZBTB1*	2.17	0.022	7.22	0.001	3.51	0.001	4.30	3.39	0.008
*IFRD1* ^b^	1.16	0.731					1.16	3.39	0.001
*BAMBI*	2.26	0.002	1.81	0.007	2.16	0.026	2.08	3.1	0.003
*IER5*	4.43	0.051	4.58	0.002	4.41	0.011	4.47	2.98	0.003
*ARMET*	2.64	0.002	2.94	0.001	3.04	0.001	2.87	2.87	0.004
*LPIN1*	2.41	0.038	2.25	0.011	2.58	0.007	2.41	2.45	0.002
*DNAJA4*	5.42	0.038	4.63	0.011	4.65	0.011	4.90	2.15	0.021
*ATF4*	2.31	0.004	2.56	0.002	2.07	0.011	2.32	2.04	0.003
*DLC1*	3.66	0.001	3.47	0.002	3.47	0.001	3.53	1.93	0.008
*ANXA5*	−2.39	0.002	−3.15	0.001	−2.99	0.001	−2.84	−2.52	0.033
*EPHX2*	−3.81	0.001	−2.28	0.001	−2.45	0.001	−2.85	−2.53	0.007
*KCNJ13*	−2.82	0.004	−2.56	0.007	−2.86	0.002	−2.75	−2.54	0.018
*CIRBP*	−4.72	0.002	−7.12	0.001	−6.84	0.001	−6.23	−2.72	0.003
*CLDN5*	−8.80	0.001	−5.49	0.001	−4.99	0.001	−6.43	−3.1	0.004
*LYPLAL1*	−3.82	0.002	−3.81	0.001	−4.22	0.001	−3.95	−3.3	0.002
*PRDM16*	−6.50	0.002	−4.17	0.001	−3.90	0.001	−4.86	−3.62	0.004

Individual gene expression (and validation). In order to assess the validity of the RNA expression data obtained by microarray, we performed RT-PCR analysis on cDNA prepared from laser-dissected CPE of the same healthy control (Br0–1) and late stage AD samples (Br5–6) as used for the microarray. We essentially followed the procedure of van Soest SS et al. (2007) [33]: In short, RT-PCR validation was done, in triplicate, on a selection of 17 genes. These genes were chosen on the basis of high expression level (>90%), significantly different expression between late stage AD and healthy control (p < 0.05) and high fold change (FC). Next, we searched for unique and efficient primers in the last 1 kb of the 3′ region the mRNA, as this area was used for the design of the oligo’s on the microarray and the microarray amplification procedure employed the poly a tail. We measured three times the expression levels of the resulting set of 17 genes by RT-PCR and calculated three times the FC and the relevant p values. Table 2 summarizes the findings and compares RT-PCRs with the outcome of the microarray. The significant difference as indicated by the microarray was confirmed for 16 of these genes, whereas the 17th gene did not amplify well in our hands in the RT-PCR, and showed unexpectedly a very low expression, perhaps due to limitations in primer design. Pearson’s correlation coefficient of the FC’s was remarkably high (*r* = 0.95) and significant (p < 0.01), confirming the gene expression results of the microarray. F C: Fold change in mRNA expression of CPE of AD (Braak 5,6) vs. healthy (Braak 0,1) donors. Pearson’s correlation coefficient between F.C. of micoarray and RT-PCR was significant (r = 0.95, p < 0.01). RT-PCR#1, RT-PCR#2, RT-PCR#3 denote three independent RT-PCRs on 7 human control samples and 7 and AD affected samples. *Some genes are represented with multiple probes on the array. If so, the value shown is the lowest P value. ^a^Some genes are represented with multiple probes on the array. If so, the value shown is the mean F.C. ^b^Our RT-PCR showed for this gene unexpectedly a very low expression, perhaps due to primer design

**Fig. 5:**
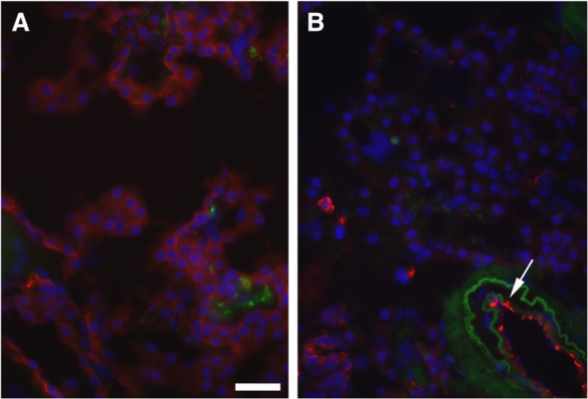
Decreased *CLDN5* immunostaining in AD affected CPE. In the healthy CP (**a**), choroid plexus epithelial (CPE) cells stained positive for CLDN5 (*red* color). In the AD tissue (**b**) the strings of CPE cells stain much less intensely for CLDN5. Note that in the same AD tissue, CLDN5 is clearly present in the blood vessel wall (*arrow*). Immunohistochemistry for CLDN5 on (6 μm thick) cryo-sections of CP from a healthy donor (A, Br0) and a donor diagnosed with AD (Br6). CLDN5 was visualized with Cy3 coupled secondary antibodies after labelling with anti-claudin 5 antibody ab53765 (Abcam). Cy3 fluorescence is shown in red. Nuclei are stained with DAPI (*blue*). The green color shows the autofluorescence that is detected in the FITC channel. This channel was added to reveal autofluorescence possibly overlapping with the Cy3 fluorescence marking the presence of CLDN5. Bar is 20 μm

We were particularly interested in the downregulation of *CLDN5* mRNA in AD affected CPE, since other investigators [36, 37] previously found variable or low expression of CLDN5 in, respectively, AD affected brain neurons and endothelial cells in the BBB of aging mice. Immunohistochemical staining of CLDN5 in our freshly frozen CPE samples showed that expression of this protein is severely reduced in the AD affected CPE (Fig. 5).

## Discussion

### AD pathophysiology in the CPE: overall features

In this study, we aimed to find consistent gene expression differences between healthy and AD-affected CPE, with corresponding functional annotations. Our gene expression data suggests that basic features of AD affected CPE cells change considerably. Obviously, whether this is (one of) the cause(s) or (one of) the consequence(s) of AD, or both, need still to be established. Nonetheless, our molecular data and functional annotations largely corroborate previous morphological, biochemical and (patho-) physiological studies on the same subject. These studies showed AD-related cellular atrophy of the CPE (due to Aβ processing and oxidative stress), impaired CSF production, clearance and efflux capacity, and altered metabolism of biomolecules by the CPE. [31, 32, 38–41]; these themes were recently reviewed by Perez-Gracia et al. (2009) and Krzyzankova et al. (2012) [42, 43].

### The unfolded protein response in AD-affected CPE cells

The most important canonical pathways upregulated in AD (Br5–6) CPE, compared to (Br0–1) CPE, indicate that CPE cells suffer from increased cellular stress: we found upregulation of the intracellular unfolded protein response, the endoplasmic reticulum stress pathway, and the protein ubiquitin pathway. The pathways E1F2 signalling and the NRF2 mediated oxidative stress response were also upregulated in AD affected CPE.

Indeed, according to the literature, AD, and other late onset neuro-degenerative disorders, are associated with stress-induced intra-neuronal accumulation of misfolded proteins. Affected neurons activate a number of protective mechanisms, such as the unfolded protein response [44], perhaps nonsense mediated RNA decay, as well as the ER-integrated stress response (ISR) [45] and the protein ubiquitin pathway [46]. In early stages of AD, the ISR temporarily decreases protein synthesis via the phosphorylation of eIF-2-alpha to reduce the overall protein load in the cell [45]. Nonetheless, prolonged stress may lead to reduction in the translation of cellular protein and, consequently, neural dysfunction and neurodegenerative disease.

Obviously, the CPE is a neuro-epithelium with multiple receptors but, unlike neurons does not have axons, dendrites or synapses. However, by similarity, we hypothesize that, in AD, Aβ induced prolonged oxidative stress impairs the major functions of the CPE (production and sink of the CSF, para-cellular transport over the BCSFB and peripheral immune cell recruitment).

### BSCFB integrity may be compromised in AD-affected CPE

We found that RNA expression of *CLDN5* was down-regulated in the AD-affected CPE cells. We confirmed the much lower presence of CLDN-5 protein in AD- affected CPE by immunohistochemistry (Fig. 5). Please note that the observed absence of CLDN5 protein may be due to either down regulation of its corresponding RNA and/or due to post-transcriptional down regulation of the CLDN5 protein, for example through interaction with the abundantly present Ubiquitin(C) complex in the (Br5–6) CPE (see Additional file 4: Figure S2, Additional file 5: Figure S3 and Fig. 5); a mechanism suggested by Mandel and co-workers in 2012 [47].

CLDN-5 is a tight junction protein and the main gatekeeper for paracellular transport over CNS-blood barriers. Our data, combined with those of the literature, suggests that the integrity of the ectodermal BCSFB barrier, and, consequently, paracellular transport may be compromised in AD. Our data corroborates and specifies previous morphological and physiological studies by Chalbot et al. (2011) and Marques et al. (2013) [14, 40], which (in-) directly point toward increased permeability of the BCSFB barrier in AD,

Interestingly, in AD-brains, the CLDN-5 protein appears to be reduced or absent in both the ectodermal BCSFB barrier (this study) as well as in the endothelial BBB barriers [36, 37]. Nonetheless, given both the structural absence of a tight neurovascular unit at the BCSFB barrier, and the specific BCSFB functions (CSF production and waste removal), we hypothesize that CLDN5 lesions at the BCSFB barrier may have a far more devastating effect on brain homeostasis.

### CSF production and sink action of AD-affected CPE

In general, two of the major functions of the CPE are CSF production and sink-action for waste-products. Literature evidence exists that the CSF pressure slowly rises in early stages of AD, and drops again in late stages of the disease [48]. Thus, if we analyse this aspect in healthy (Br0–1) CPE and AD affected (Br5–6) CPE, we expect to get a complicated and mixed molecular picture.

Boassa and Yool 2005 previously implicated the AQP1 protein in CSF production [49]. We found that the CPE expression of AQP1 remains high in both healthy and AD-affected CPE (Additional file 10: Table S5), which confirms that CSF pressure (changes) can only partly explained by the action of this protein [13]. We previously published a molecular model of aqueous humor and CSF production [50, 51] and we identified a number of relevant genes whose expression was also significantly regulated in our current study: These include ATP1B3 (up in network 1); ATPASE (up; network 3); KCNJ13 (down; network 18); AQP6 (down; network 22), KCNE4 (up; network 15) and phosphates (up in network 4 and down in 1). As we expected, we could not deduce a clear picture which molecular mechanism(s) in the CPE cause(s) CSF pressure, in part, to rise and drop again.

Nonetheless, two canonical pathways in our data set may be important for CSF pressure regulation (i.e. upregulated aldosterone signaling) and CPE sink action (i.e. downregulated glutathione-mediated detoxification). Aldosterone is a steroid hormone produced in the adrenal cortex. It plays a major role in Na+, K+, H+ and water homeostasis through transcriptional and translational regulation of electrolyte transport. Aldosterone is well known for its role in blood pressure regulation, but was recently also implicated in regulation of CSF pressure [52]. Glutathione-mediated detoxification is a general detoxification pathway, where the first step is catalysed by glutathione transferases (GST). Glutathione binds to toxic electrophilic chemicals, forming conjugates, which are exported from the (CPE) cell, and, perhaps, removed from the brain. Consequently, since the glutathione-mediated detoxification pathway is downregulated, our data may indicate that part of the sink action of the AD-affected CPE is compromised.

### Further specific observations in AD-affected CPE

We analysed the CPE expression of several genes previously implicated in AD, including APP, APOE, PSEN1, PSEN2, CLU, ABCA7 and TTR. Most of these genes are moderately to highly expressed in the CPE. However, no statistical significant gene expression difference(s) could be found for these genes between healthy and affected CPE samples. Since the TTR expression was very high, and reached the saturated feature range in both healthy or AD- affected CPE on several microarrays, we could not formally establish a potential difference in TTR expression between healthy and affected CPE. Our data indicate that the AD-affected CPE, despite partial loss of cellular integrity, produces at least the same or more TTR compared to non-affected CPE (Additional file 10: Table S5 and Additional file 2: Figure S1).

We observed in the Top 10 genes of the highest and lowest gene expression (fold)-changes, between (Br5–6) CPE and (Br0–1) CPE, a number of genes with unknown function (for example C6orf58, C10orf107). We also found genes with more or less known function, but (yet) unknown relationship with AD (for example BRCA2, FABP4, LSMEM1, ETNPPL, CELLSR1, SERPINC1, ZB1TB1). Finally, we found a number of genes with more or less known function for which a possible relationship with AD may be obvious, or has been described: For example, upregulation of the heat shock proteins HSPA1, HSPA6 and DNAJB1/Hsp40 reflects increasing cellular CPE stress; the prion protein 2 (PRND) was downregulated. Several authors reported conflicting (genetic association) results of the possible relationship between PRND (SNPS) and AD [53].

The literature labelling of our molecular (Br5–6) CPE vs (Br0–1) CPE networks (see Additional files) yielded several AD relevant observations. While their exact role in the pathogenesis of AD is frequently not (fully) known, a number of striking observations are briefly highlighted below. Literature labelling did not implicate potential human choroid plexus failure in relation to folate processing. However, we did observe changes in several transport entities, such as copper and iron and vitamin C transport or homeostasis. We also observed upregulation of the vitamin D receptor gene (VDR; network 4) in AD-affected CPE. Interestingly, Durk et al. [54] found recently that vitamin D3 reduces cerebral amyloid Beta-accumulation and improved cognition in AD mouse models. In addition, Lee YH et al. [55] found, in a meta-analysis, genetic association between two Vitamin D Receptor (VDR) SNPS and AD. We found upregulation of both the acetylcholine (ACH) receptor (network 2) and of the ACH esterase inhibitor CYP3A4 (network 5). Indeed, several authors showed previously that AD is accompanied by a severe functional deficit in the cholinergic network, which affects both neurotransmission and immune reactions [56]. We also found upregulation of glutharedoxin (GLRX; GRX) (network 2). GLRX is essential to maintain a reduced intracellular environment under conditions of increased oxidative stress. Interestingly, Arodin and co-workers [57] previously measured an increased release of GRX protein in the CSF of AD patients. We observed upregulation of the gonadotropin release hormone (GNRHR; network 5) gene. Our data are in line with the findings of Nuruddin et al. (2014) [58] who found elevated mRNA levels of GNRH and its receptor GNRHR in plaque bearing AD mouse models. Next, we noticed upregulation of Wiskott-Aldrich syndrome family member 1 (WASF1; WAVE). Already in 2009, Takata and co-workers found co-aggregation of WAVE protein with modified hyper-phosphorylated tau in neurofibrillary tangles in both AD affected brain and 3xTg AD mouse models [59]. We found upregulation of the MTORC1 gene expression (network 7) in AD affected CPE. Our data corroborate the findings of several authors, who found that the neuroprotective MTORC1 pathway is activated in human AD [60] as well as in relevant mouse models [61]. We also observed downregulation of CaMK, which is most likely involved in phosphorylation of Tau in AD [62]. Finally, we noticed that expression of one or more genes of the G protein coupled receptor group (GpCr) was downregulated (network 18). GPCRs mediate the proteolysis of APP and degradation of amyloid beta. Vice versa, amyloid supposedly beta perturbs GPCR function [63].

## Conclusions

In AD affected CPE, we found and annotated specific cellular changes probably due to increased oxidative stress, such as the unfolded protein response, E1F2 and NRF2 signalling and the protein ubiquitin pathway. Further, our data suggests that the AD affected BCSFB barrier becomes more permeable due to downregulation of “gatekeeper” CLDN5. Finally, our data also predicted down regulation of the glutathione mediated detoxification and the urea cycle in the AD CPE, which suggest that the CPE sink action may be impaired. Literature labelling of our functional molecular networks confirmed multiple previous (molecular) observations in the AD literature and revealed many new ones. We conclude, on the basis of our molecular and functional annotation data, that CPE failure in AD exists.

Combining our data with those of the literature, we suggest the following, more or less chronological order of events taking place at the CPE in AD: increased amyloid burden on CPE; increased oxidative stress; despite continuous and high TTR production: decrease of CPE capability to handle increased amyloid burden; (pro-) inflammatory signalling; intracellular ubiquitin involvement; remodelling of adherens and tight junctions (CLDN5) causing increased permeability, and, finally, cellular atrophy and barrier disintegration. Thus, in AD, the brain barriers, especially the BCSFB, remodel, and may become leaky. The BCSFB increasingly loses its function to support CNS homeostasis (CSF production, macrophage recruitment and paracellular transport). Taken together, the available data strongly support the concept that CP failure contributes to AD pathogenesis.

## Availability of supporting data

Following MIAMI guidelines, all the raw data is available at the GEO database Submission nr GSE61196 [NCBI tracking system #17129928]. Other supporting data is available at the Additional files associated with this manuscript.

## Additional files

Additional file 1: Tabel S1. Contains characteristics of donor eyes used. (XLS 25 kb) Additional file 2: Figure S1. Cryostate sections of choroid plexus of a control patient (Braak stage 0) showing double staining for the choroid plexus epithelium marker transthyretin (TTR), (B, Cy3, red) and the tight junction protein 1 (Zo-1), (C, alexa 647, green). Nuclei are stained with DAPI (A, blue). D (merged) shows the double staining of choroid plexus epithelium. Bar is 25 μm. (TIFF 3280 kb) Additional file 3: Table S2. Contains compilations of lists of (disease) genes previously implicated in relevant disease. The entries are used to label the molecular network in Additional file 4: Figure S2 and Additional file 5: Figure S3. (XLS 190 kb) Additional file 4: Figure S2. Upregulated genes in AD (Br5–6). Molecular networks are presented which were generated by the core analysis of the Ingenuity knowledge database (see also Methods section and www.ingenuity.com for explanation and details). The symbols (with Genbank Gene name) in the network come from: input genes (Additional file 6: Table S3: upregulated genes in AD Br5–6) see also description below (Additional file 5: Figure S3). (PDF 17491 kb) Additional file 5: Figure S3. Downregulated genes in AD Br5–6. The input genes Additional file 8: Table S4) are bioinformatically translated to proteins and the symbols are colored. Another set of genes/proteins is inserted by the knowledgedatabase to construct the most likely networks; the symbols are white. Different types of symbols exist (for explanation see www.ingenuity.com: transporters, strucural proteins, secreted proteins, etc). The symbols are connected by solid lines and dotted lines. These denote, respectively, direct physical/functional and indirect interaction between the symbols/genes/proteins. These interactions are, via the knowledgde database, derived form wet lab experiments in the literature, and from big data stored in curated public databases (GEO, etc). The most likely networks are constructed by the knowledge database, taken all available data into account of human, mouse, rat and in vitro model experiments. Finally, once the networks are constructed, specific entries in the networks can be labelled, by hand of the user, to specifically label molecules wihch are involved in sopecific diseases or biological process of interest. In this way, the (predicted) molecular enviroment of entries of interest can be explored. (ZIP 10804 kb) Additional file 6: Table S3. Contains the specifications of genes upregulated in CPE Br(5–6) compared to CPE Br(0,1). (XLSX 26 kb) Additional file 7: Figure S4. In this file a standard output of the most established and most simple (linear) representation of biology (canonical pathways) are given on the basis of input data (in this case statistically significant BH corrected upregulated genes in AD; Additional file 6: Table S3). The knowledge database recognizes enriched biological themes (for example: unfolded protein response etc.) in the input data compared to a random distribution of genes over all relevant biological themes. In the figure, the y-axis denotes statistical significance levels, the horizontal axis contains biological themes. The bars indicate the statistical significance for that particular theme. The orange horizontal line gives the significance level P = 0.05. The orange irregular line follows the ratio (number of molecules in particular theme/relevant number of genes under consideration). For further details, see www.ingenuity.com. (JPEG 76 kb) Additional file 8: Table S4. Contains the specifications of genes downregulated in CPE Br(5–6) compared to CPE Br(0,1). (XLSX 31 kb) Additional file 9: Figure S5. See description for Additional file 7: Figure S4, but now the input used are the down-regulated genes in CPE AD (5–6) Additional file 8: Table S4). (JPEG 56 kb) Additional file 10: Table S5. Presents the gene expression in our datasets of a number of previously known AD disease genes. (XLSX 12 kb)
