# Supplementary figures and images for: Gene expression and functional annotation of human choroid plexus epithelium failure in Alzheimer’s disease
Source: BMC Genomics. 2015 Nov 16;16:956. doi: 10.1186/s12864-015-2159-z (PMC4647590; doi:10.1186/s12864-015-2159-z)

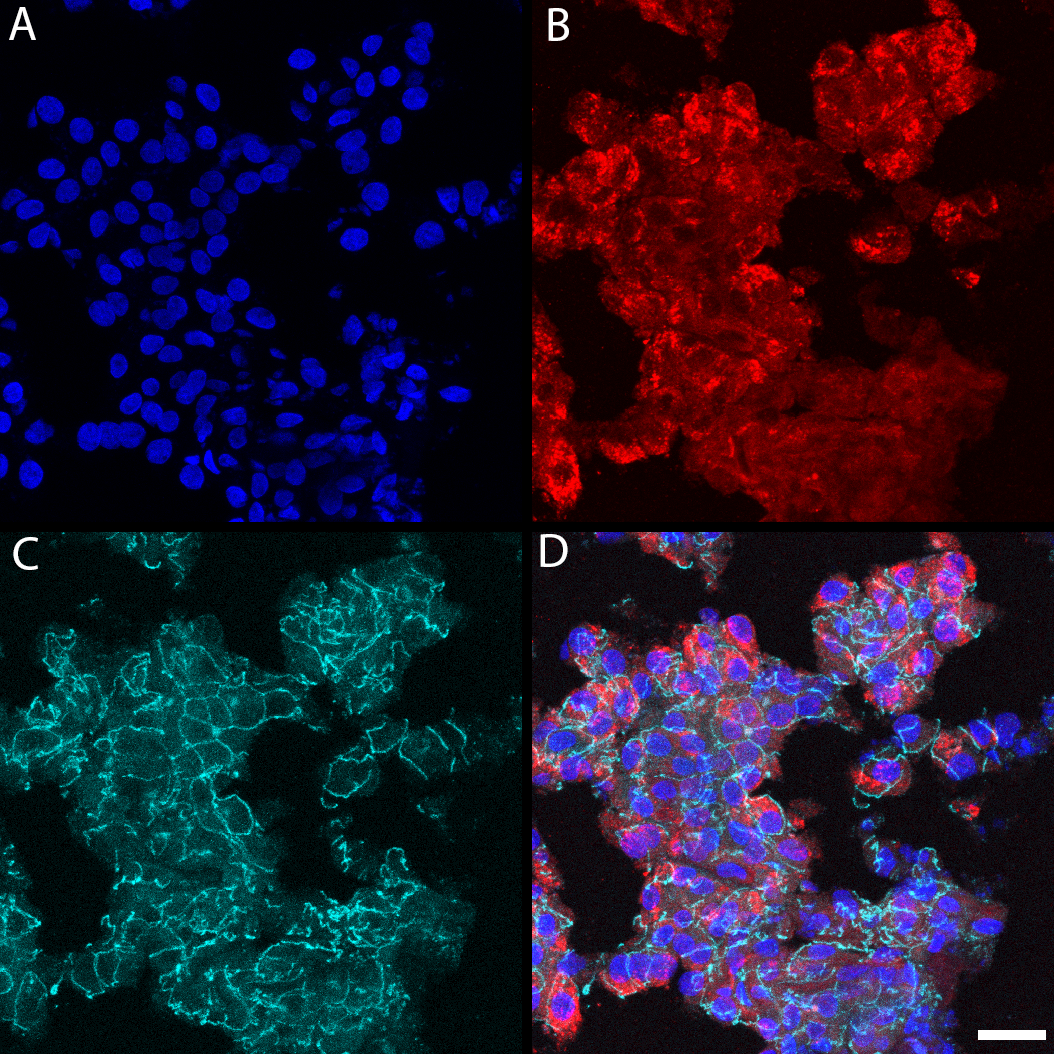

Supplement: Additional file 2: Figure S1. — Cryostate sections of choroid plexus of a control patient (Braak stage 0) showing double staining for the choroid plexus epithelium marker transthyretin (TTR), (B, Cy3, red) and the tight junction protein 1 (Zo-1), (C, alexa 647, green). Nuclei are stained with DAPI (A, blue). D (merged) shows the double staining of choroid plexus epithelium. Bar is 25 μm. (TIFF 3280 kb) [file 12864_2015_2159_MOESM2_ESM.tif]

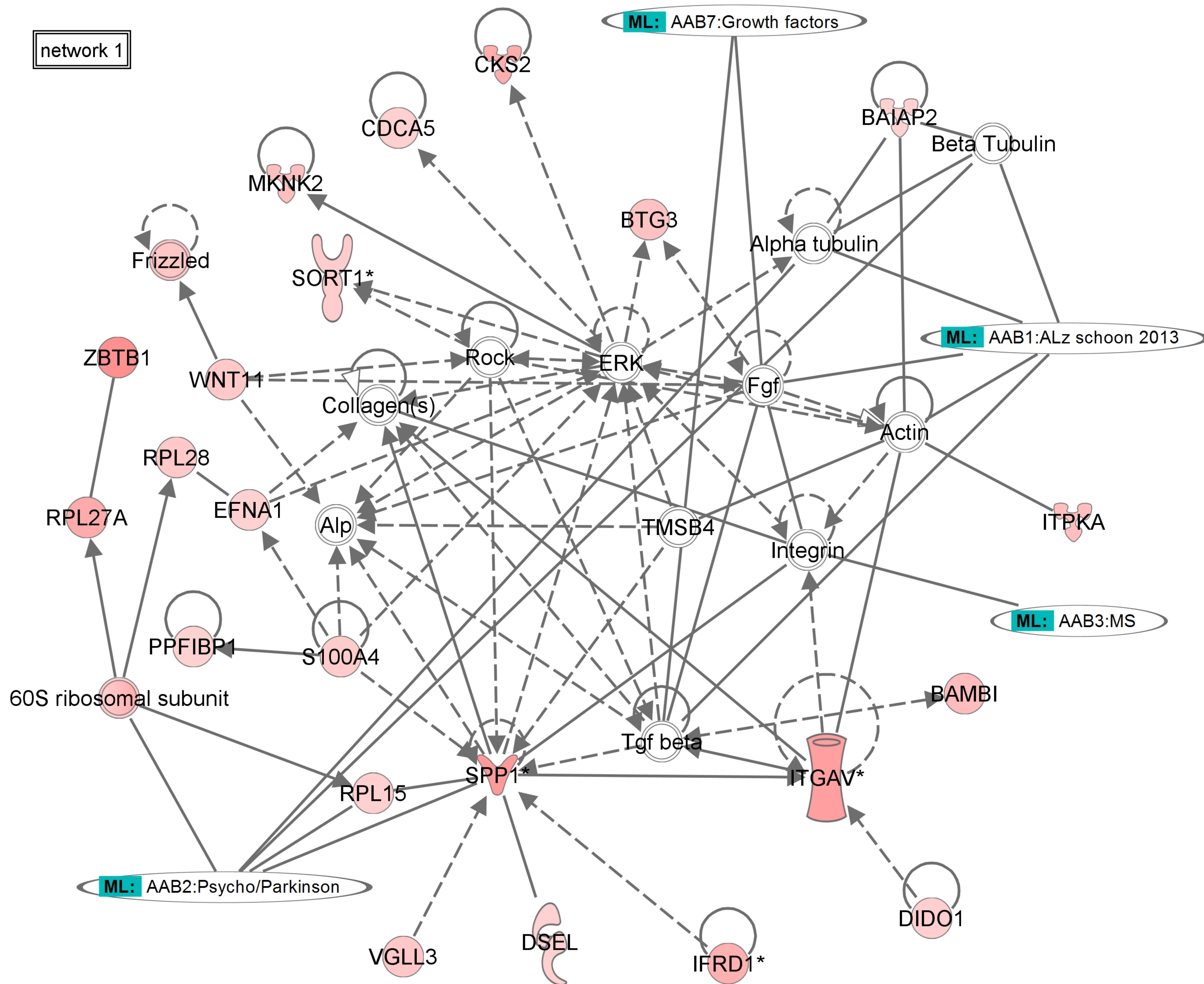

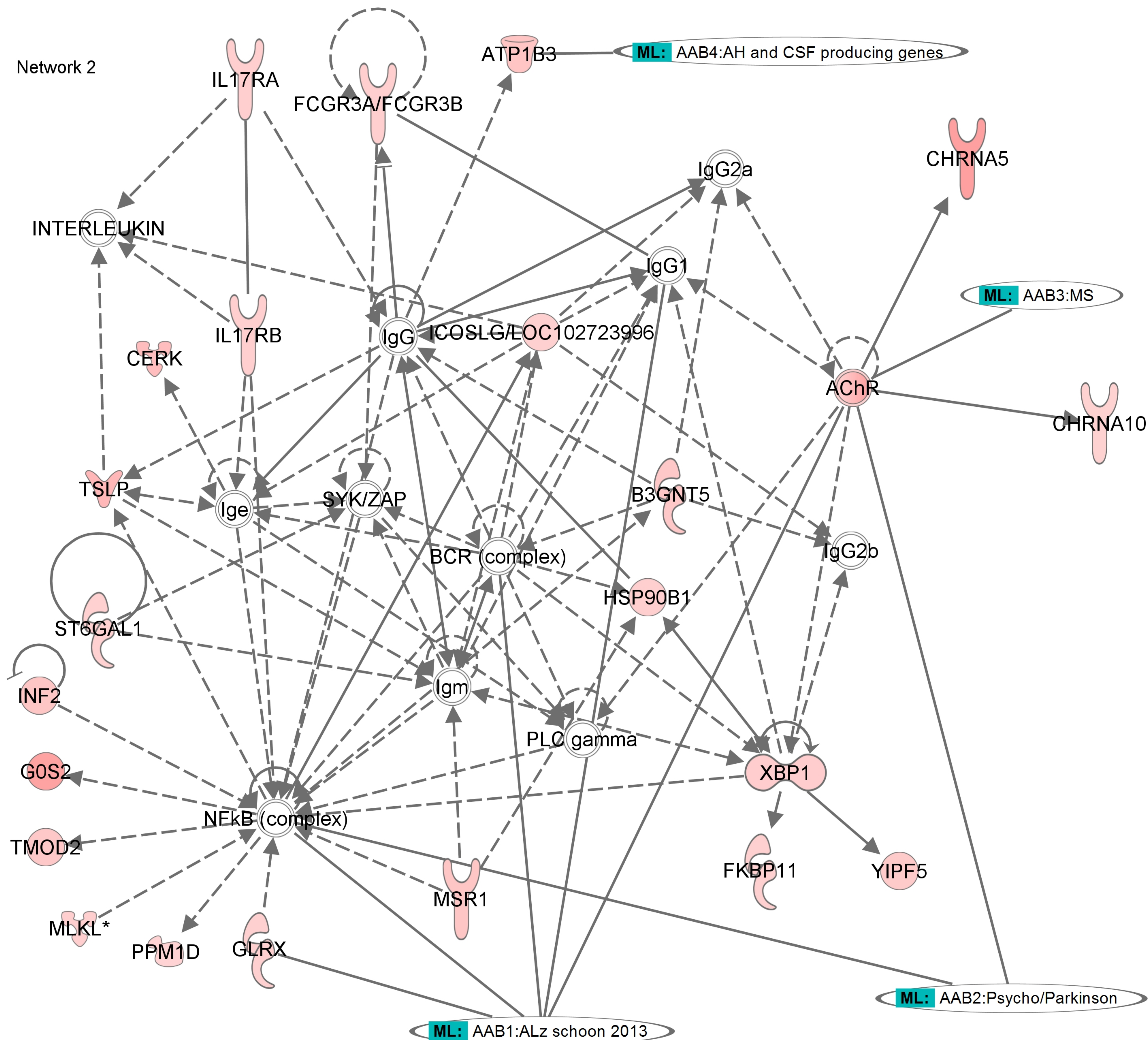

Network 3

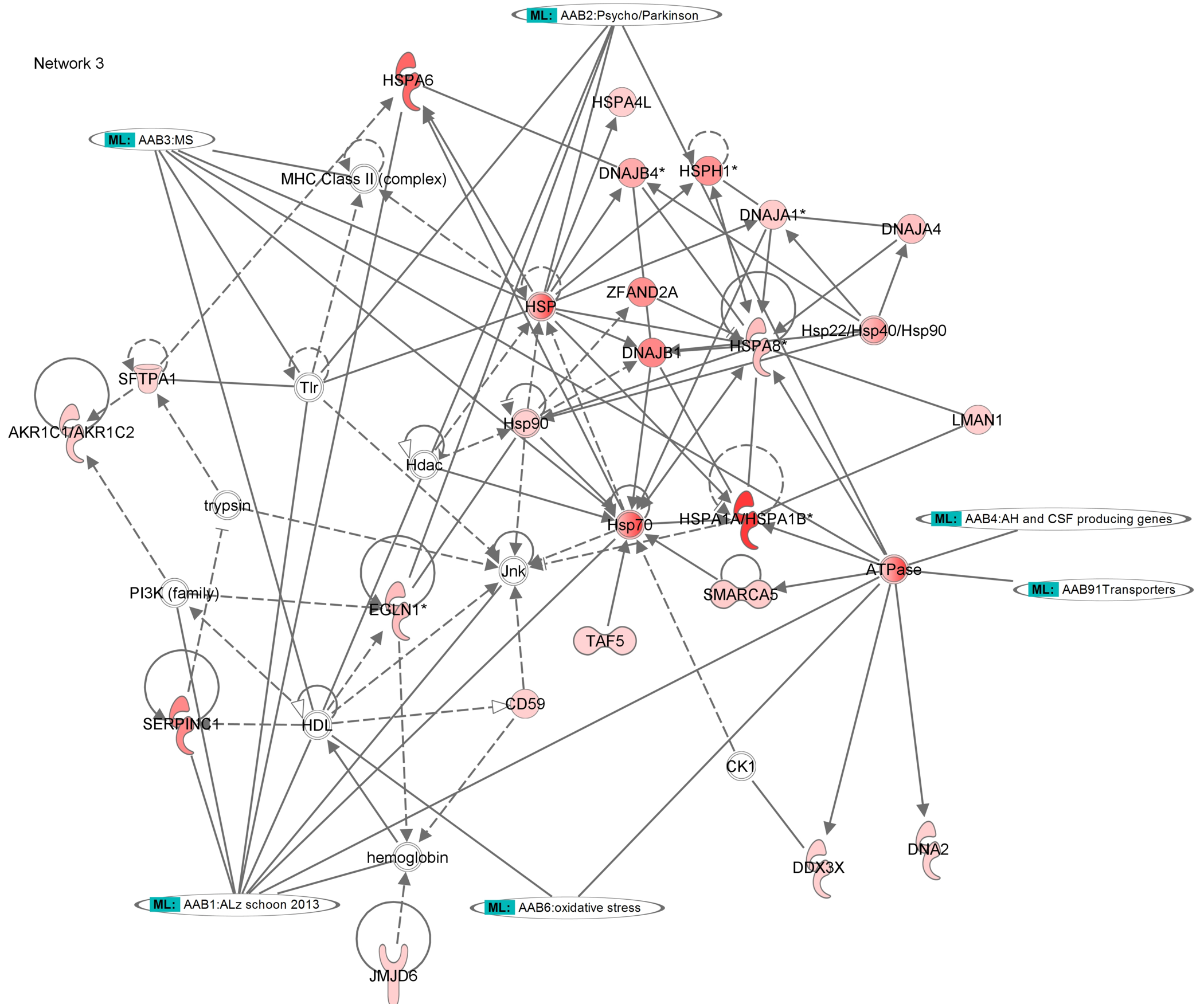

Network 4

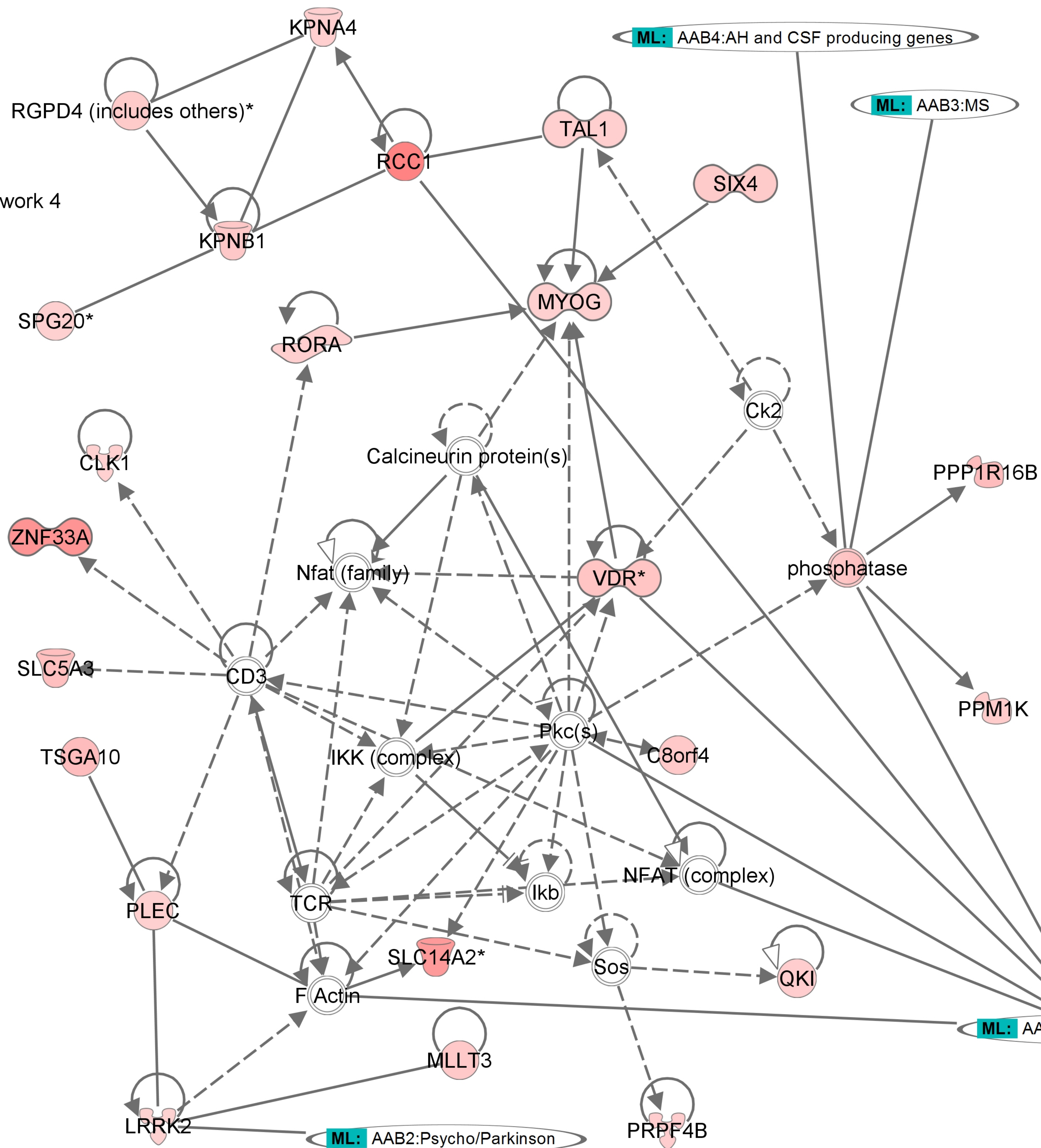

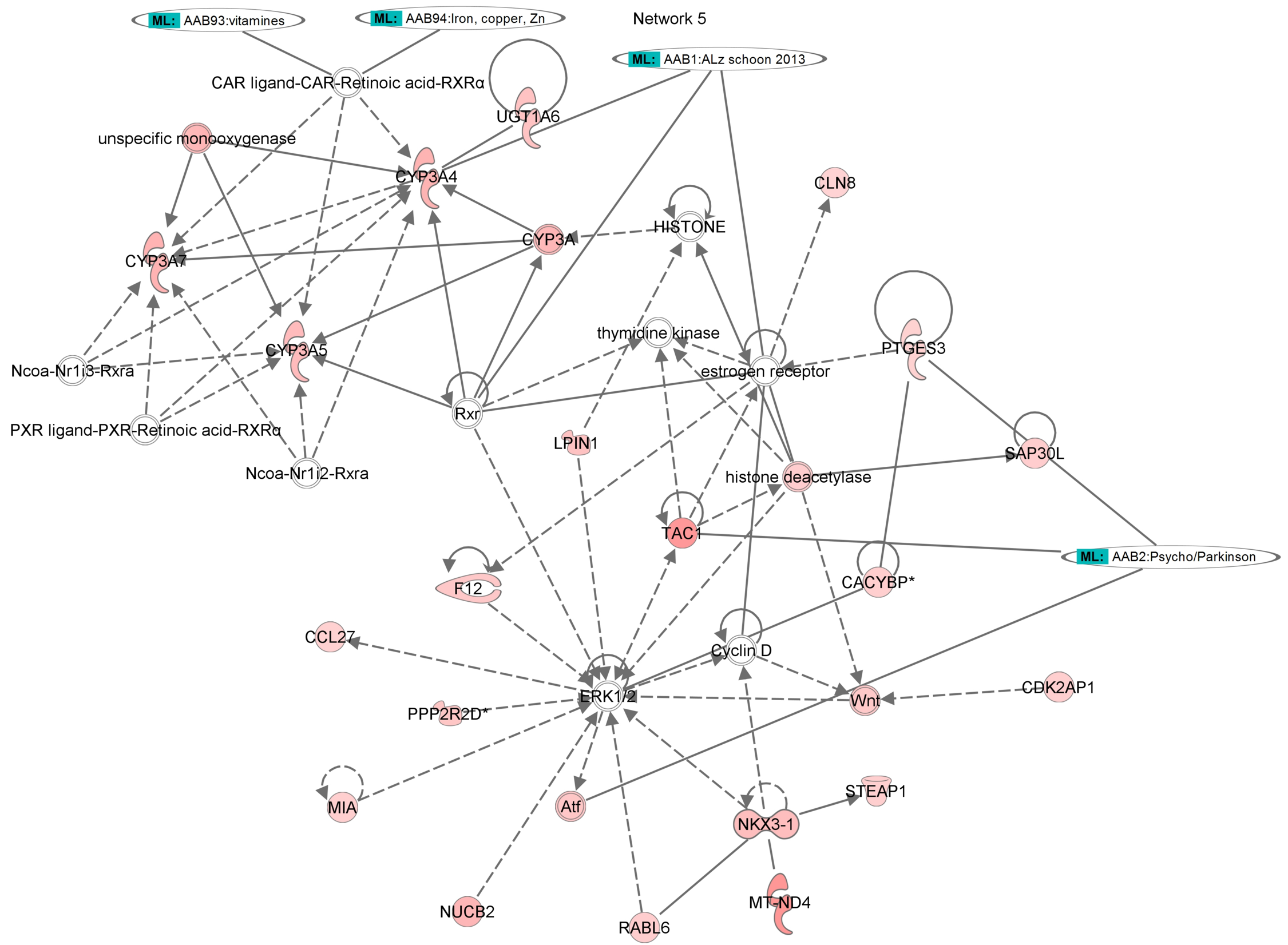

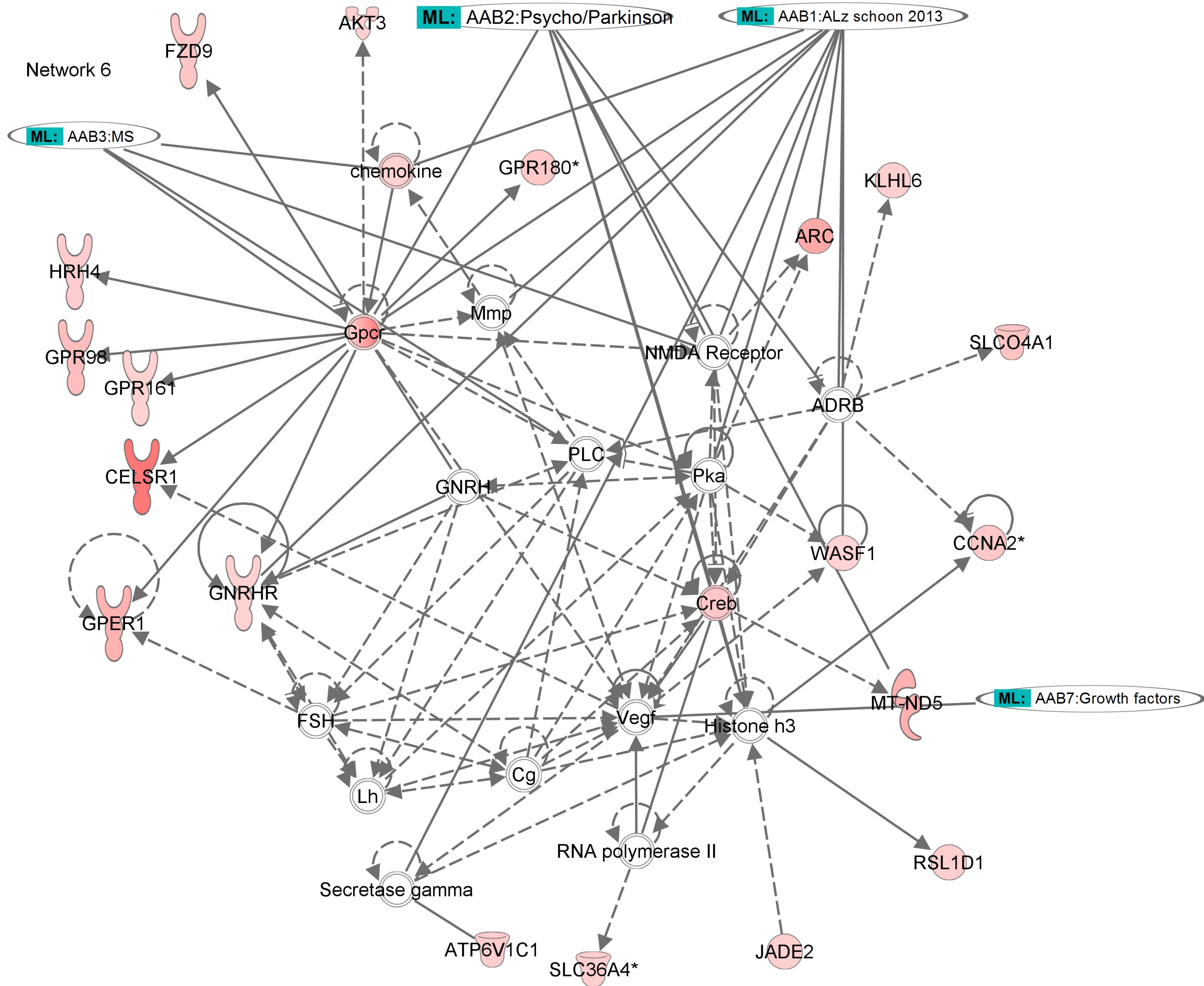

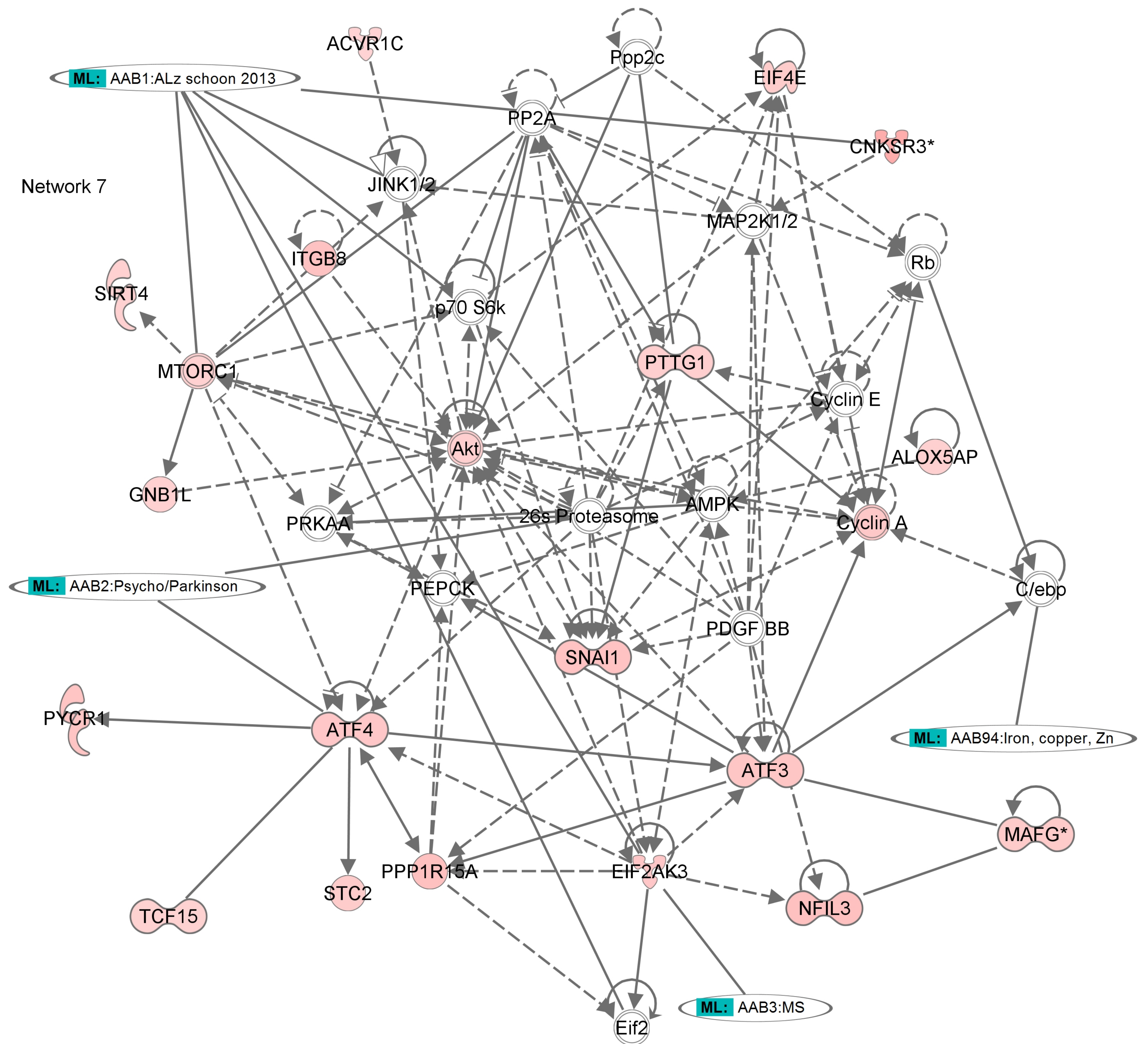

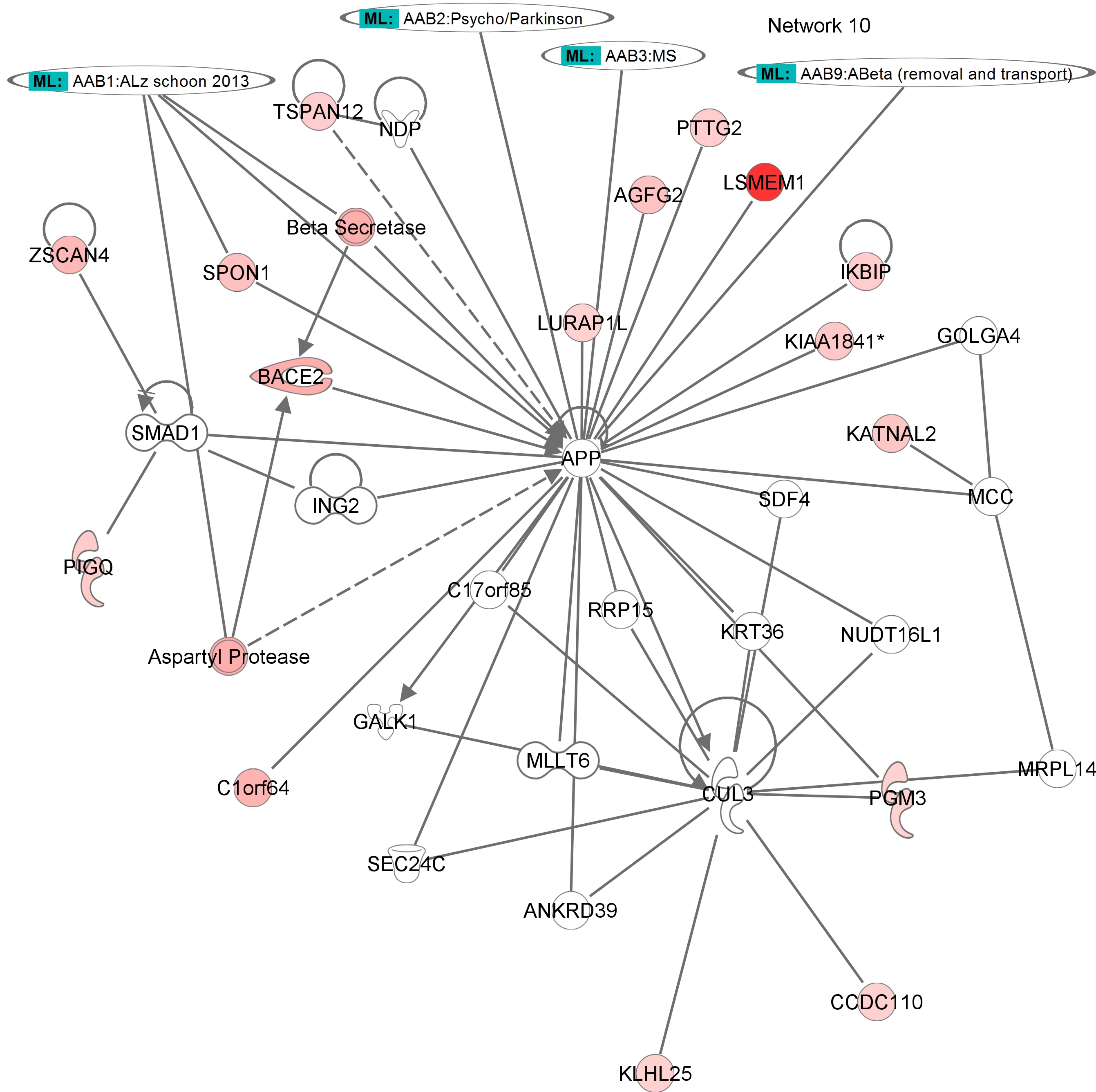

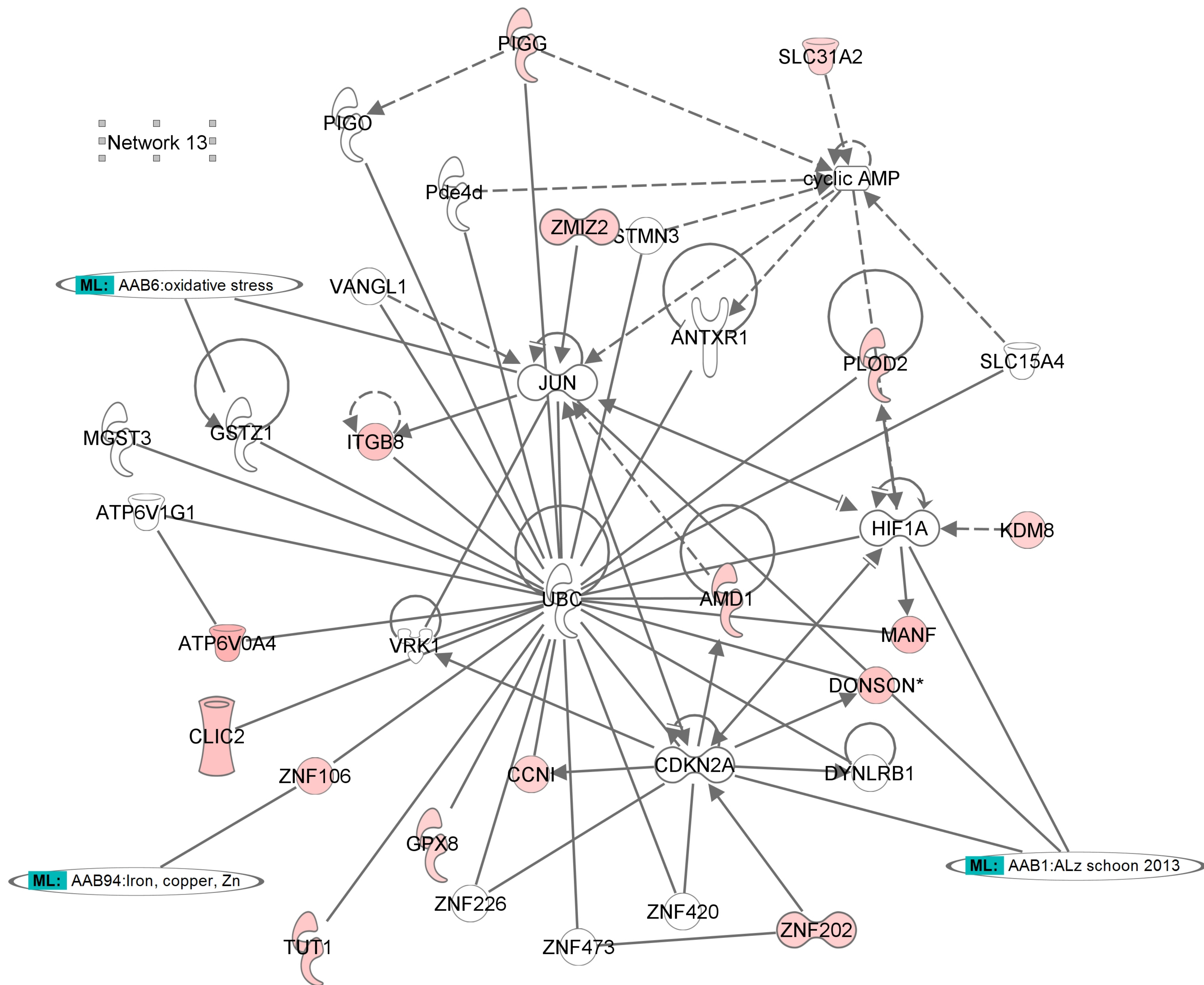

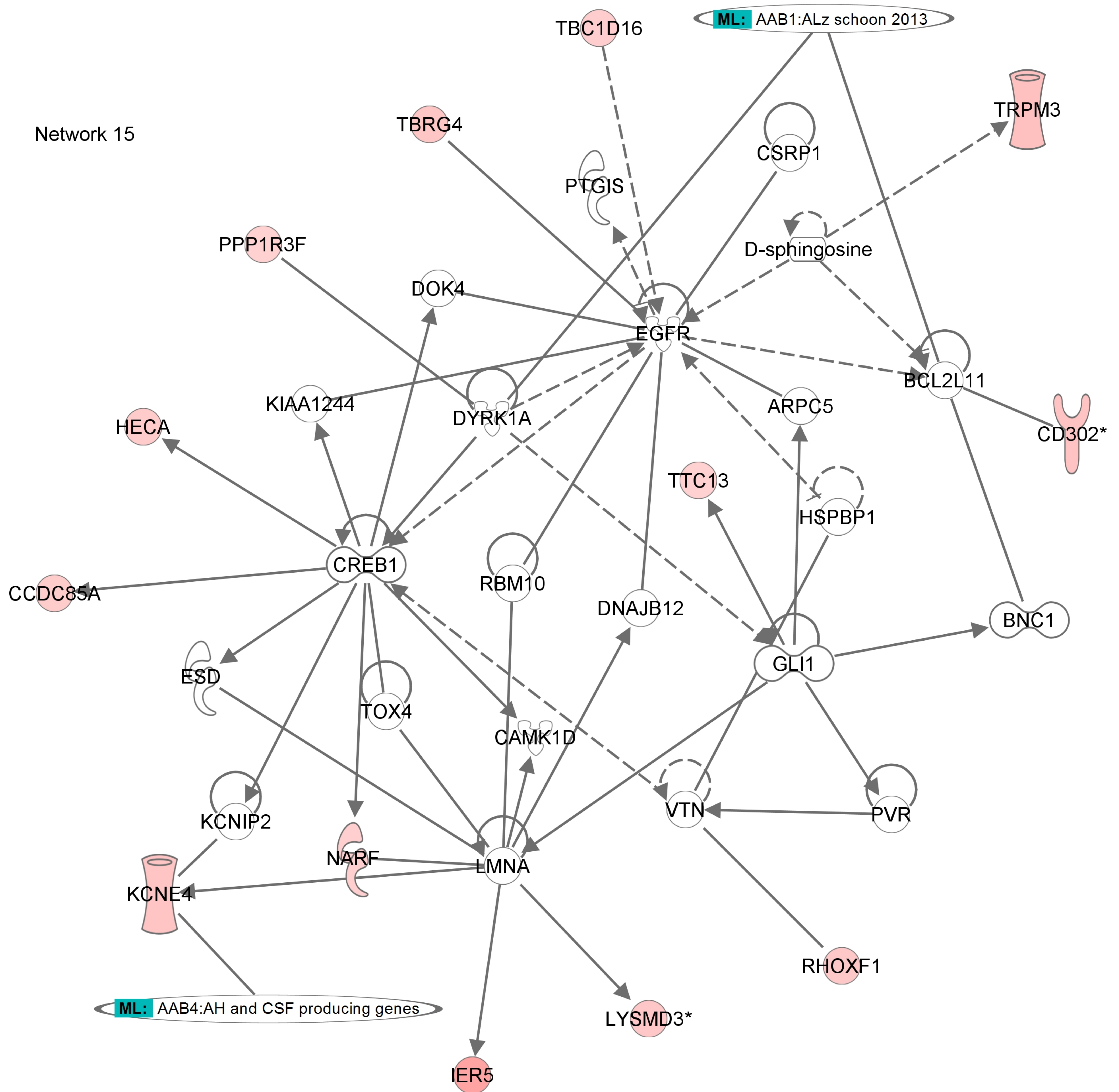

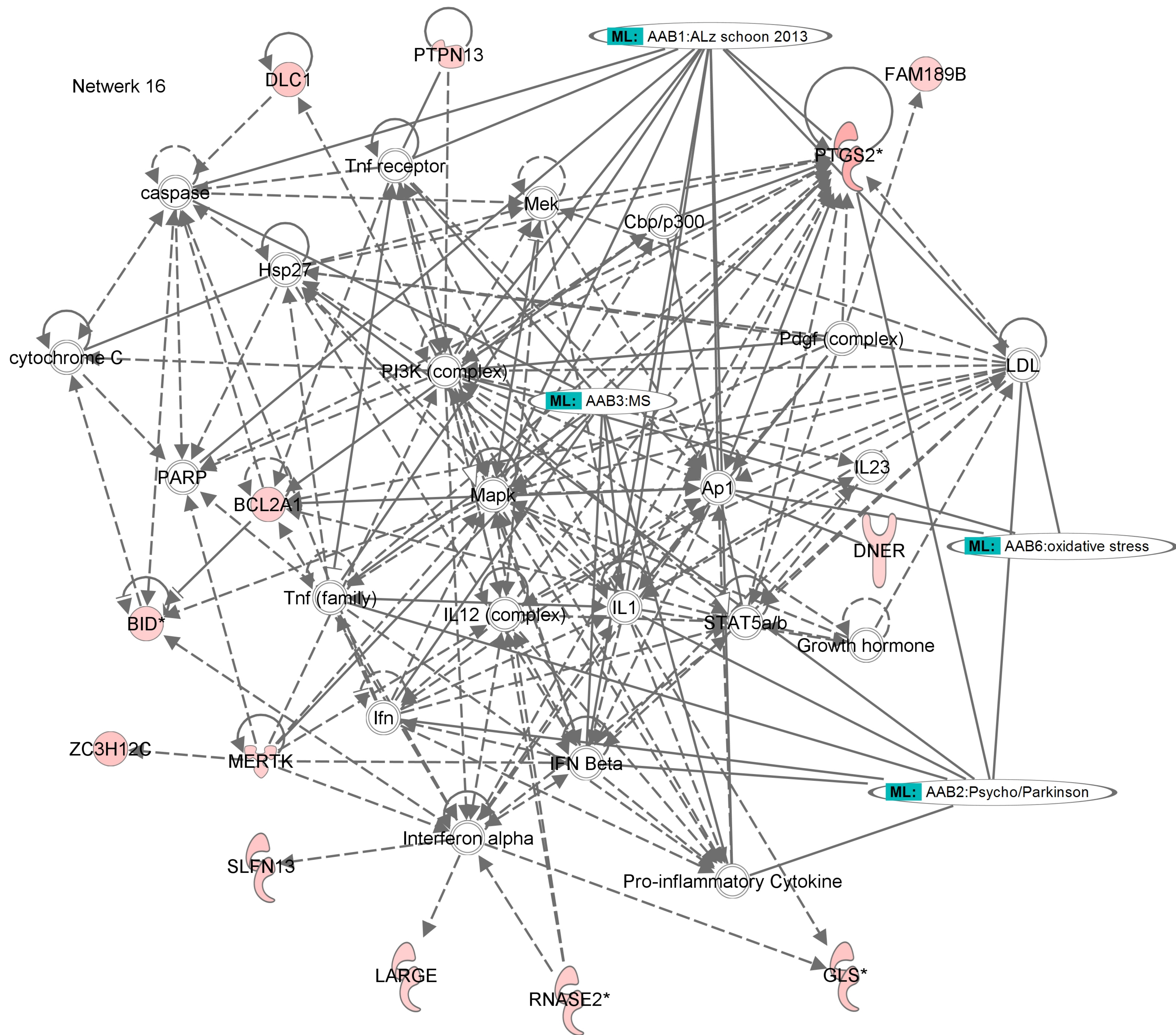

Supplement: Additional file 4: Figure S2. — Upregulated genes in AD (Br5–6). Molecular networks are presented which were generated by the core analysis of the Ingenuity knowledge database (see also Methods section and www.ingenuity.com for explanation and details). The symbols (with Genbank Gene name) in the network come from: input genes (Additional file 6: Table S3: upregulated genes in AD Br5–6) see also description below (Additional file 5: Figure S3). (PDF 17491 kb) [file 12864_2015_2159_MOESM4_ESM.pdf]

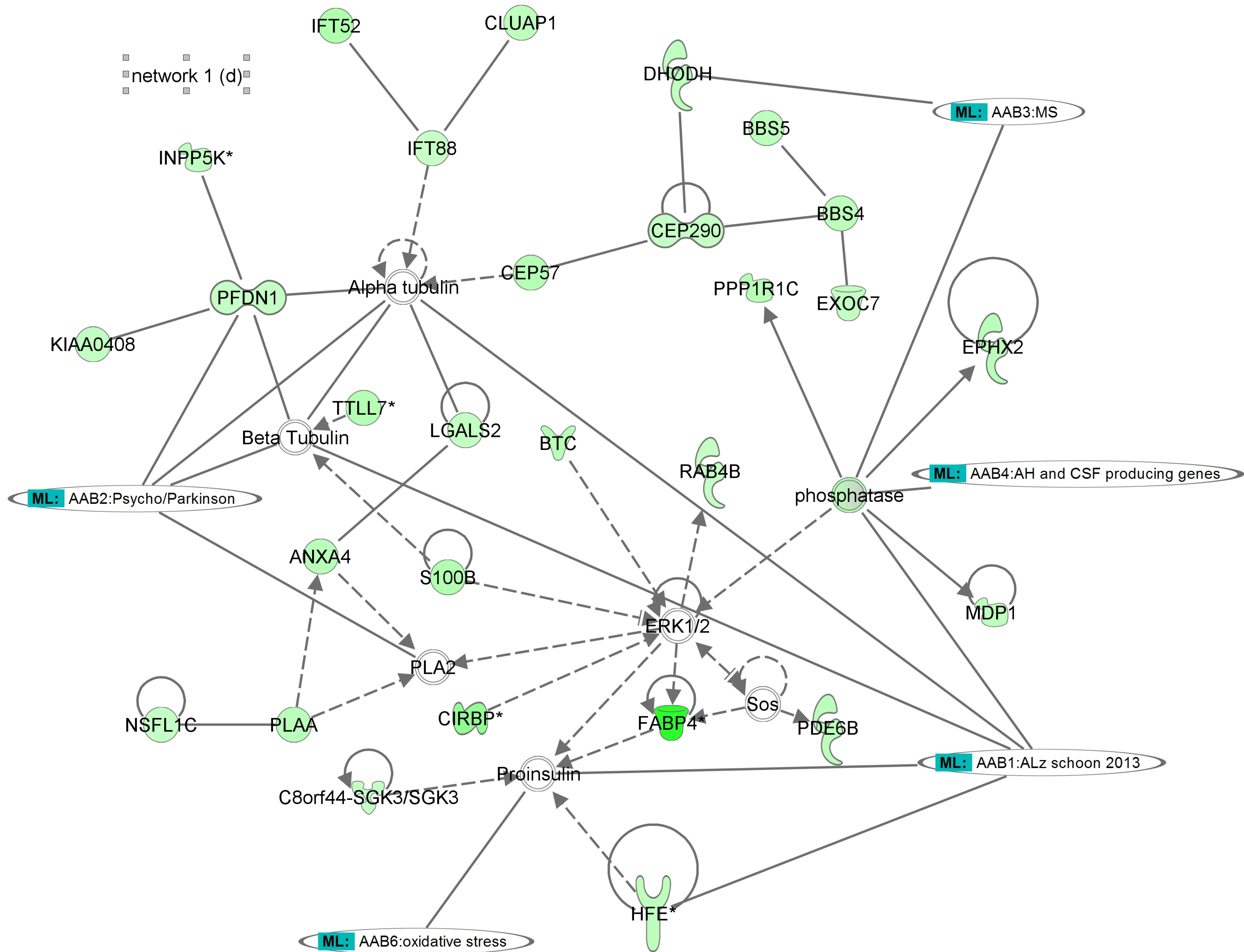

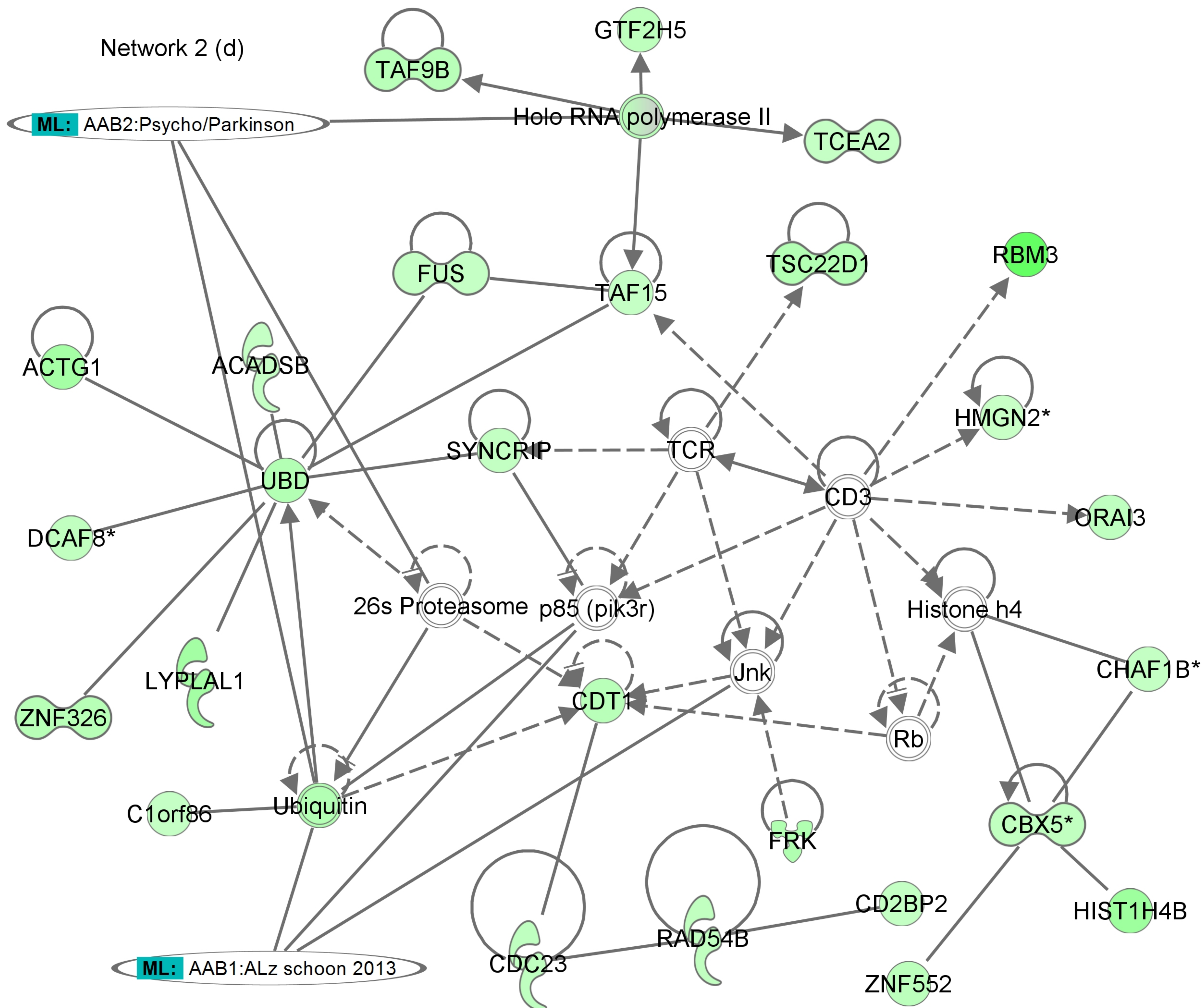

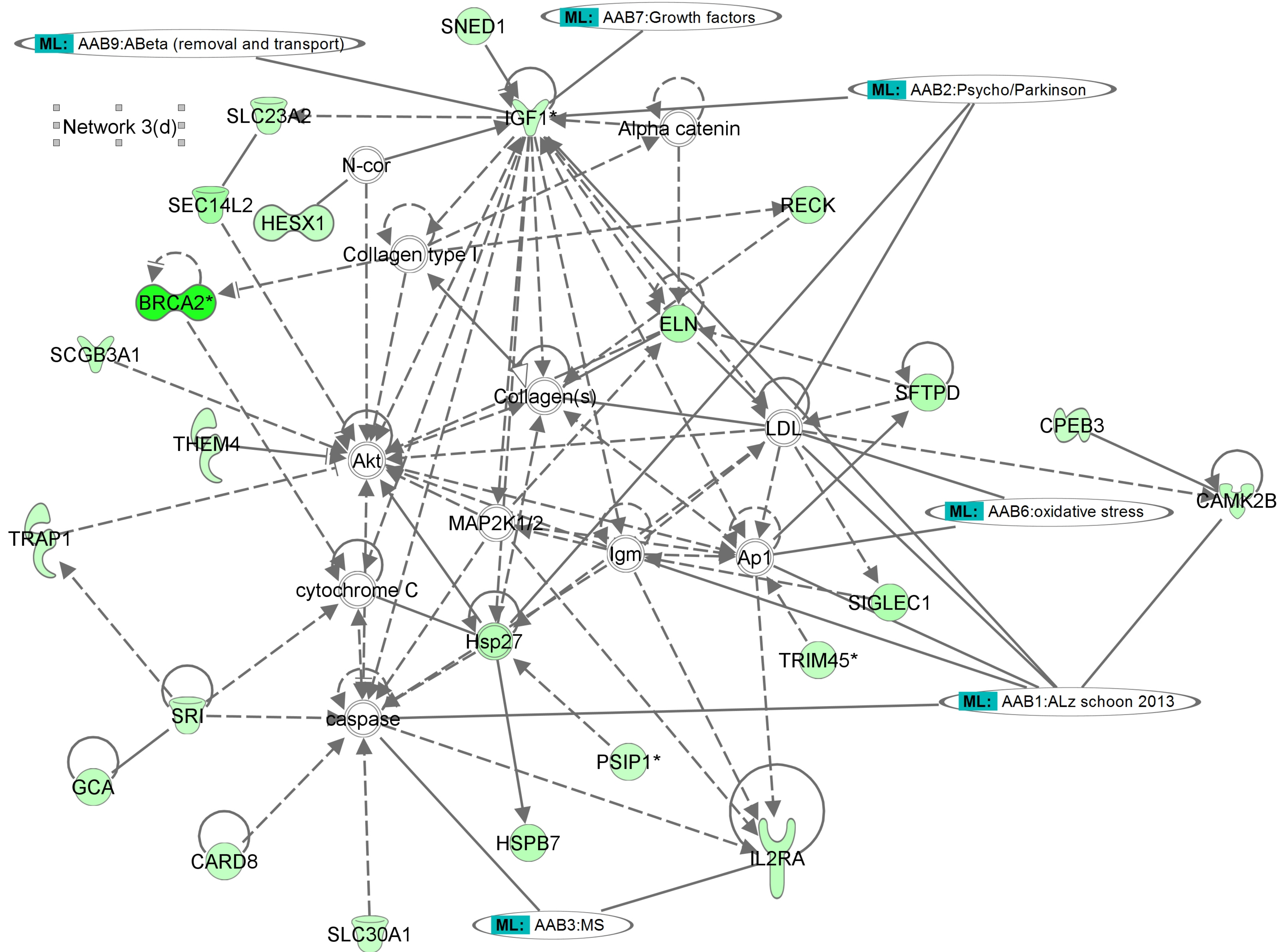

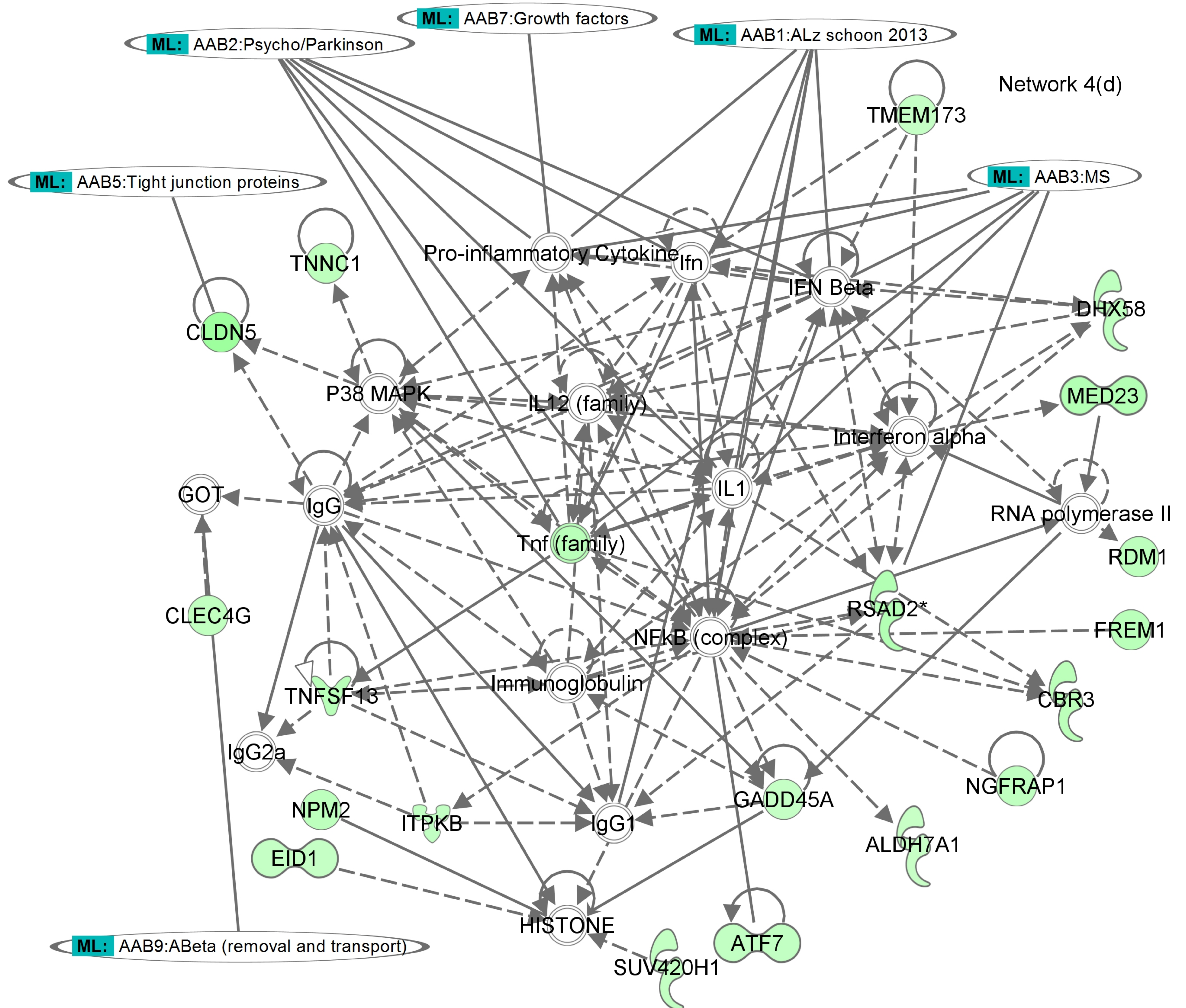

Network 6 (d)

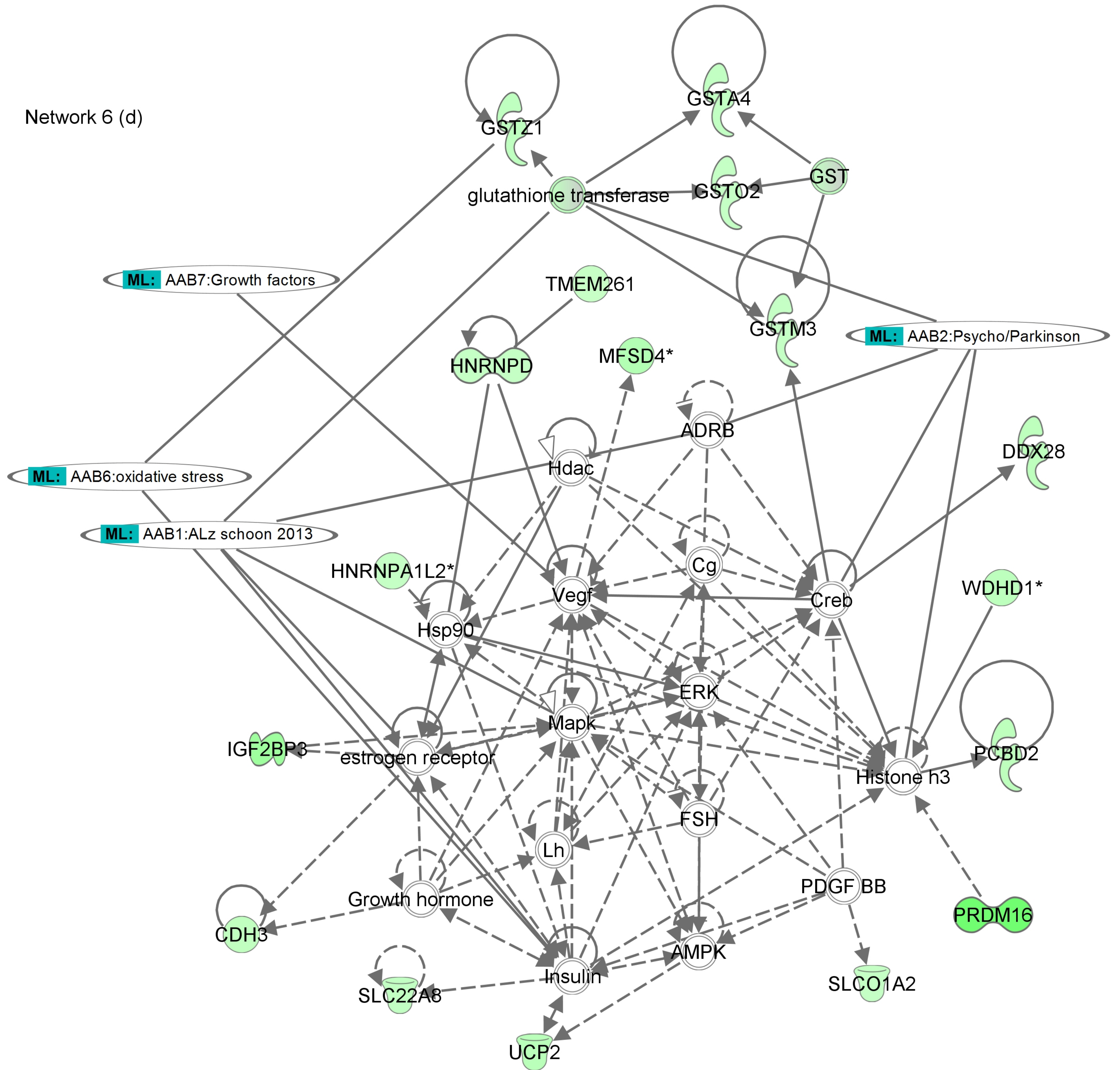

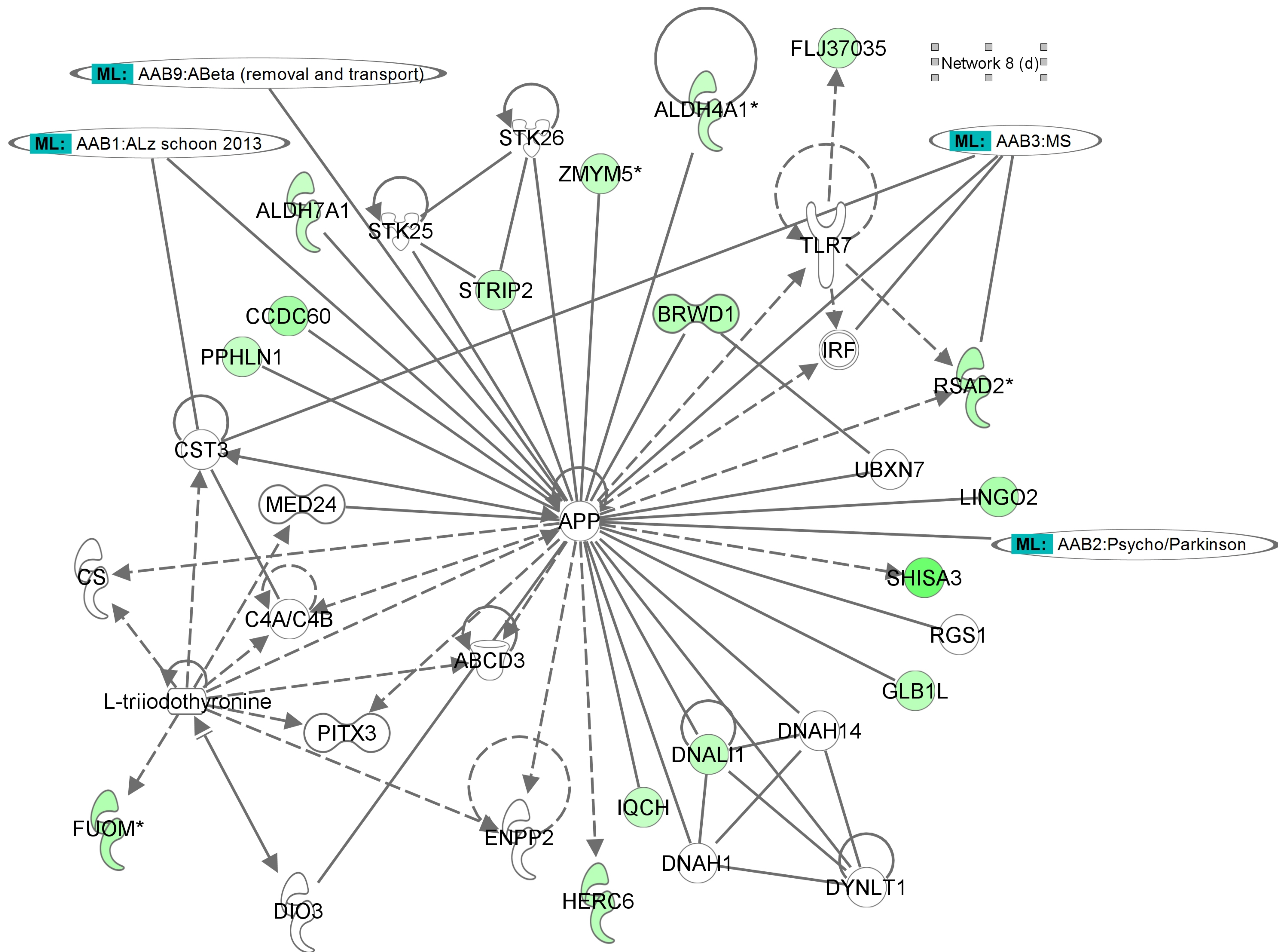

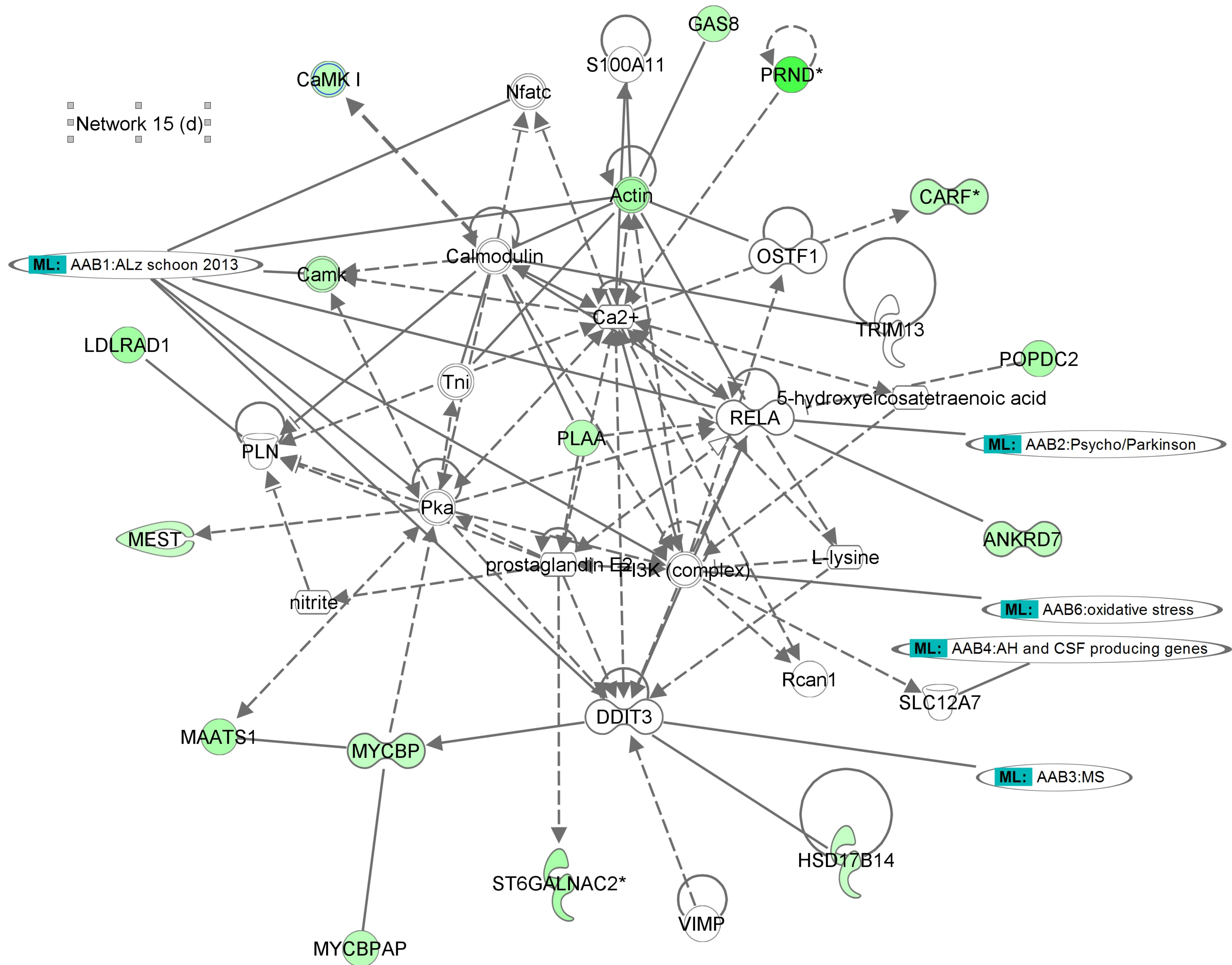

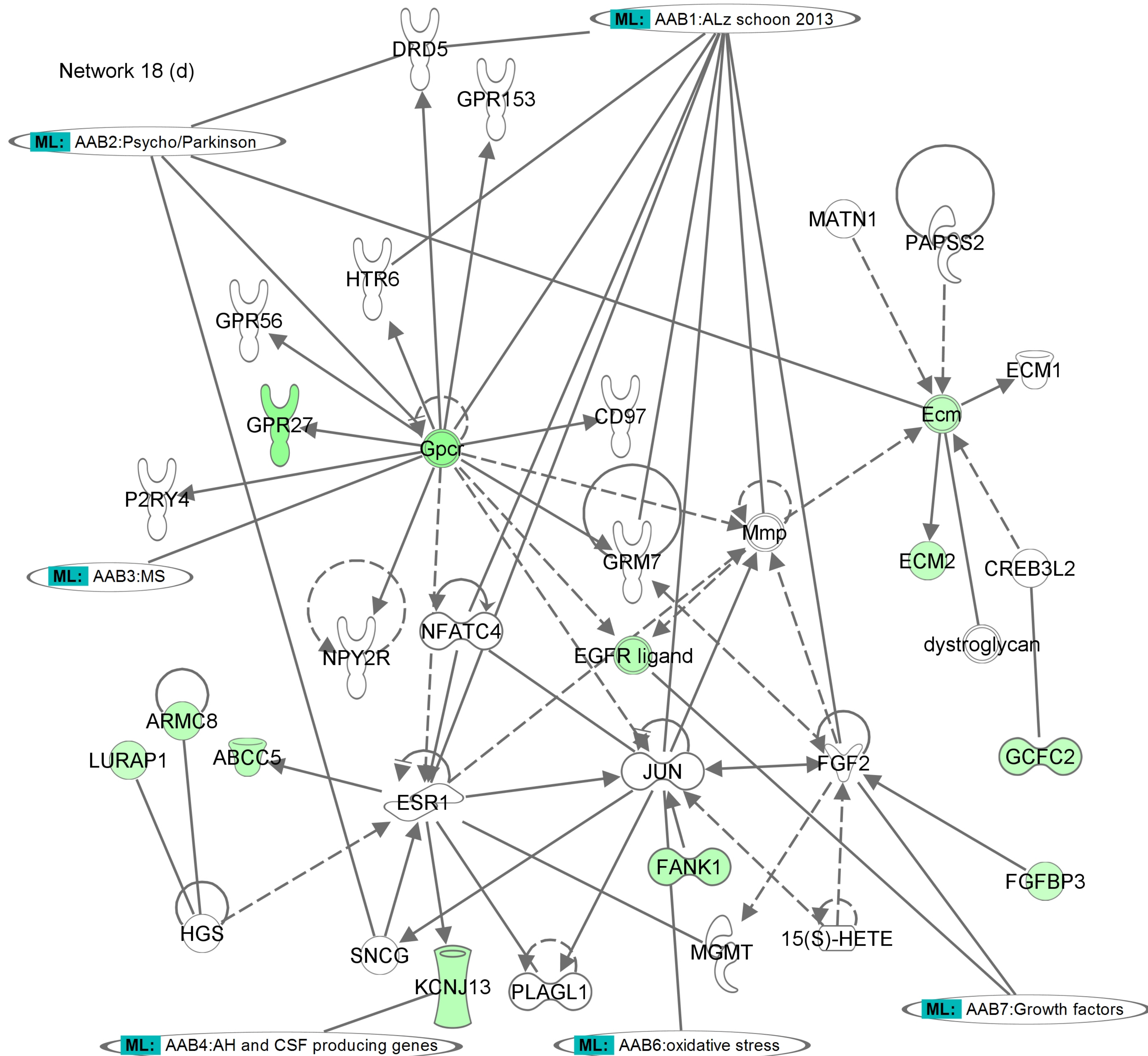

# Path Designer Network 22 down and ann

■ ■ ■  
■ Network 22 ■  
■ ■ ■

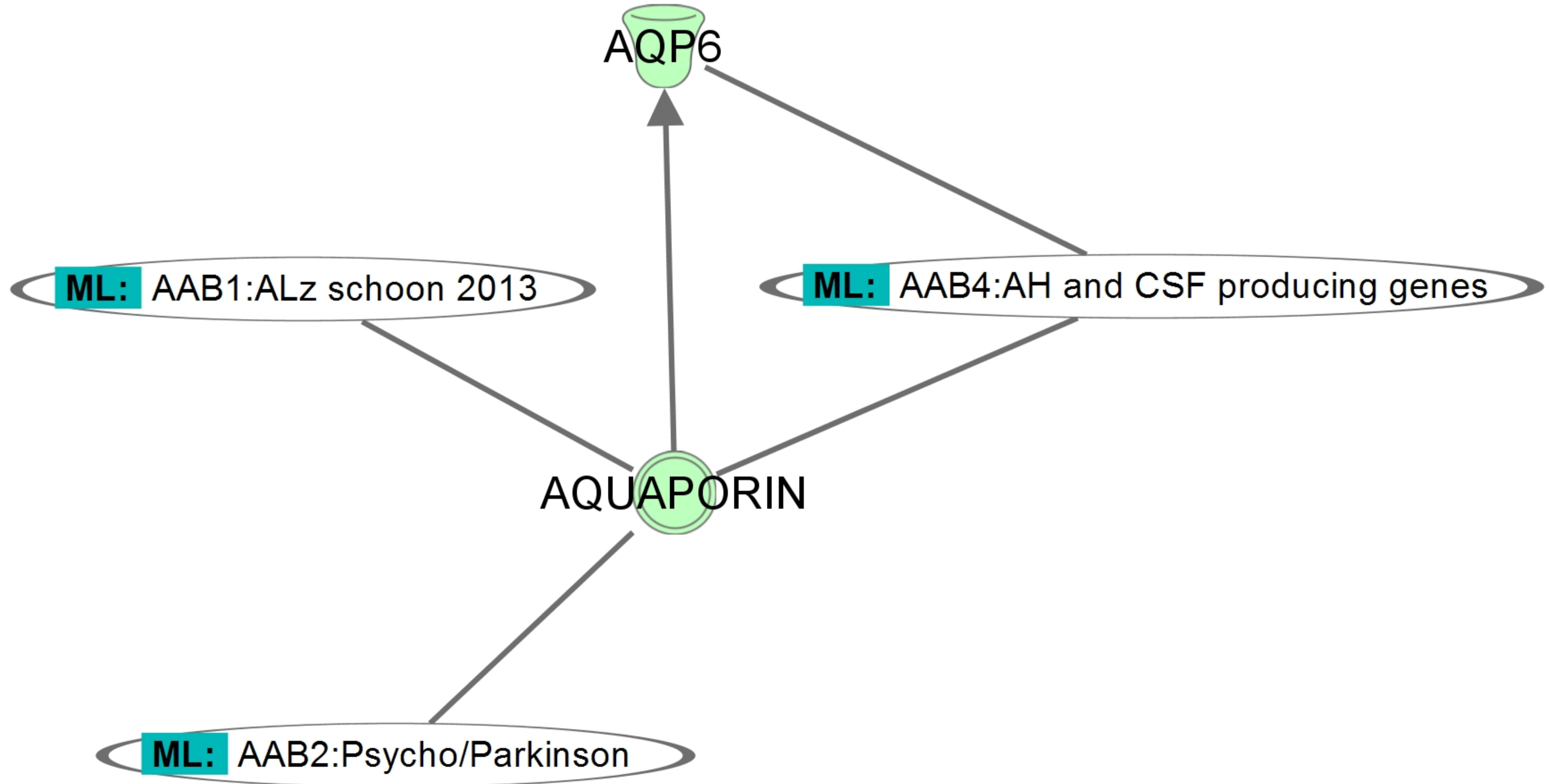

Supplement: Additional file 5: Figure S3. — Downregulated genes in AD Br5–6. The input genes Additional file 8: Table S4) are bioinformatically translated to proteins and the symbols are colored. Another set of genes/proteins is inserted by the knowledgedatabase to construct the most likely networks; the symbols are white. Different types of symbols exist (for explanation see www.ingenuity.com: transporters, strucural proteins, secreted proteins, etc). The symbols are connected by solid lines and dotted lines. These denote, respectively, direct physical/functional and indirect interaction between the symbols/genes/proteins. These interactions are, via the knowledgde database, derived form wet lab experiments in the literature, and from big data stored in curated public databases (GEO, etc). The most likely networks are constructed by the knowledge database, taken all available data into account of human, mouse, rat and in vitro model experiments. Finally, once the networks are constructed, specific entries in the networks can be labelled, by hand of the user, to specifically label molecules wihch are involved in sopecific diseases or biological process of interest. In this way, the (predicted) molecular enviroment of entries of interest can be explored. (ZIP 10804 kb) [file 12864_2015_2159_MOESM5_ESM.zip › NewSupplementary figure 3 networks down.pdf]

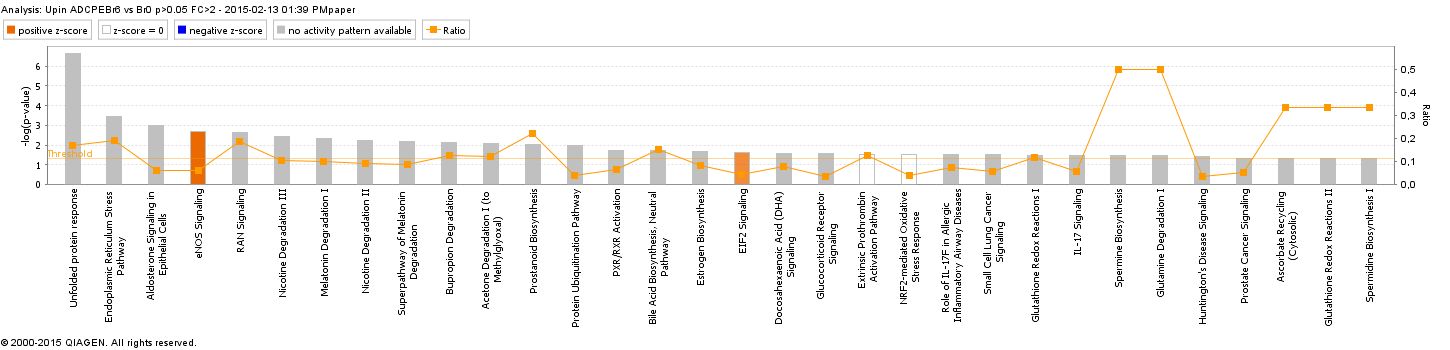

Supplement: Additional file 7: Figure S4. — In this file a standard output of the most established and most simple (linear) representation of biology (canonical pathways) are given on the basis of input data (in this case statistically significant BH corrected upregulated genes in AD; Additional file 6: Table S3). The knowledge database recognizes enriched biological themes (for example: unfolded protein response etc.) in the input data compared to a random distribution of genes over all relevant biological themes. In the figure, the y-axis denotes statistical significance levels, the horizontal axis contains biological themes. The bars indicate the statistical significance for that particular theme. The orange horizontal line gives the significance level P = 0.05. The orange irregular line follows the ratio (number of molecules in particular theme/relevant number of genes under consideration). For further details, see www.ingenuity.com. (JPEG 76 kb) [file 12864_2015_2159_MOESM7_ESM.jpg]

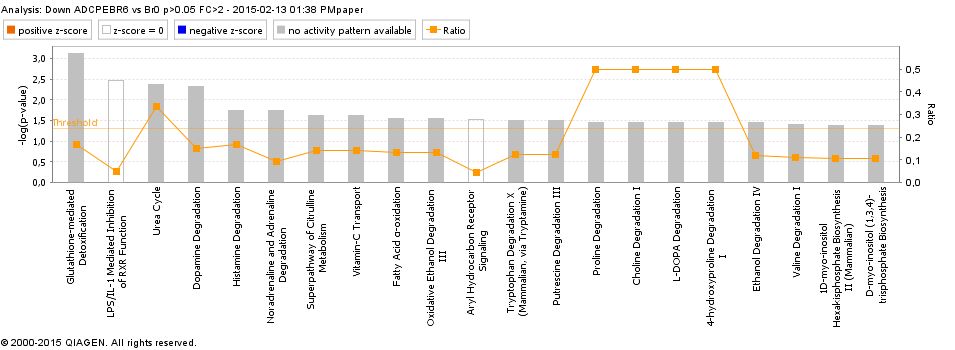

Supplement: Additional file 9: Figure S5. — See description for Additional file 7: Figure S4, but now the input used are the down-regulated genes in CPE AD (5–6) Additional file 8: Table S4). (JPEG 56 kb) [file 12864_2015_2159_MOESM9_ESM.jpg]
